# Hybrid allele-specific ChIP-seq analysis identifies variation in brassinosteroid-responsive transcription factor binding linked to traits in maize

**DOI:** 10.1186/s13059-023-02909-w

**Published:** 2023-05-08

**Authors:** Thomas Hartwig, Michael Banf, Gisele Passaia Prietsch, Jia-Ying Zhu, Isabel Mora-Ramírez, Jos H. M. Schippers, Samantha J. Snodgrass, Arun S. Seetharam, Bruno Huettel, Judith M. Kolkman, Jinliang Yang, Julia Engelhorn, Zhi-Yong Wang

**Affiliations:** 1 Department of Plant Biology, Carnegie Institution for Science, 260 Panama Street, Stanford, CA 94305 USA; 2 Present Address: Heinrich-Heine University, Universitätsstraße 1, Düsseldorf, NRW 40225 Germany; 3 Max Planck Institute for Plant Breeding Research, Carl-von-Linné-Weg 10, Cologne, NRW 50829 Germany; 4 Leibniz-Institute of Plant Genetics and Crop Plant Research (IPK) Gatersleben, Corrensstraße 3, Seeland, SA 06466 Germany; 5 Department of Ecology, Evolution, and Organismal Biology, Iowa State University, 339A Bessey Hall, Ames, IA 50011 USA; 6 School of Integrative Plant Science, Plant Pathology and Plant-Microbe Biology Section, Cornell University, 413 Bradfield Hall, Ithaca, NY 14853 USA; 7 Department of Agronomy and Horticulture, University of Nebraska-Lincoln, 363 Keim Hall, Lincoln, NE 68583 USA

**Keywords:** Allele-specific, Transcription factor, ChIP-seq, Brassinosteroid, Regulatory network, Functional variation

## Abstract

**Background:**

Genetic variation in regulatory sequences that alter transcription factor (TF) binding is a major cause of phenotypic diversity. Brassinosteroid is a growth hormone that has major effects on plant phenotypes. Genetic variation in brassinosteroid-responsive cis-elements likely contributes to trait variation. Pinpointing such regulatory variations and quantitative genomic analysis of the variation in TF-target binding, however, remains challenging. How variation in transcriptional targets of signaling pathways such as the brassinosteroid pathway contributes to phenotypic variation is an important question to be investigated with innovative approaches.

**Results:**

Here, we use a hybrid allele-specific chromatin binding sequencing (HASCh-seq) approach and identify variations in target binding of the brassinosteroid-responsive TF ZmBZR1 in maize. HASCh-seq in the B73xMo17 F1s identifies thousands of target genes of ZmBZR1. Allele-specific ZmBZR1 binding (ASB) has been observed for 18.3% of target genes and is enriched in promoter and enhancer regions. About a quarter of the ASB sites correlate with sequence variation in BZR1-binding motifs and another quarter correlate with haplotype-specific DNA methylation, suggesting that both genetic and epigenetic variations contribute to the high level of variation in ZmBZR1 occupancy. Comparison with GWAS data shows linkage of hundreds of ASB loci to important yield and disease-related traits.

**Conclusion:**

Our study provides a robust method for analyzing genome-wide variations of TF occupancy and identifies genetic and epigenetic variations of the brassinosteroid response transcription network in maize.

**Supplementary Information:**

The online version contains supplementary material available at 10.1186/s13059-023-02909-w.

## Background

Linking genetic variation to phenotypic variation is the ultimate goal of genetic and genomic studies. While most studies focus on variation in protein-coding sequences, it recently became clear that variation in transcriptional regulation is a major cause of phenotypic diversity [1]. One of the emerging themes of genome-wide association studies (GWAS) is that a large fraction of sequence polymorphisms that are statistically associated with phenotypic variation are located in non-genic portions of the genome [2]. In maize, it is estimated that up to 50% of natural phenotypic variation is caused by non-coding variants [3–5]. This influence on trait variation makes such variants ideal targets for bio-engineering efforts to improve yield-relevant traits. However, GWAS often identifies a relatively large region that contains many variants, such as single-nucleotide polymorphisms (SNPs), or small insertions, or deletions (INDELs). Unlike variations in coding sequences, the functional impact of non-coding variations cannot be reliably predicted based on the sequence. Variation in non-coding regions influences phenotypes by altering transcription factor binding and gene expression [6, 7]. Therefore, experimental analysis of variation in transcription factor binding at genome scale would provide molecular functions of non-coding variants. When combined with GWAS, the specific variation in transcriptional regulation (alterations in *cis*-element function) can be linked to variation in traits. Efforts such as the human ENCODE project have developed genome-wide maps of variants affecting TF binding for mammalian systems and used them to link *cis*-regulation to key traits such as disease risk [8]. Currently, we lack such genomic maps of variants affecting TF binding in crops.

Plant growth and biomass accumulation are controlled mainly by hormones, among which brassinosteroid (BR) has a major growth-promoting effect and diverse functions in development and physiology in all higher plants. BR acts through a receptor kinase (BRI1) signaling pathway to activate the BZR1 family of TFs. Research in *Arabidopsis* has identified thousands of BZR1 target genes, which mediate BR regulation of cell elongation, development of roots, shoots, organ boundaries, reproductive development, and photomorphogenesis and disease resistance [9].

Genetic studies indicate that BR acts through a similar signal transduction mechanism and regulates similar and unique developmental processes in crops such as rice and maize. Similar to *Arabidopsis*, BR-deficient maize mutants are extreme dwarfs with severely reduced organ sizes. In maize, BR plays an additional unique role in sex determination, and BR-deficient maize develops female florets and sets seeds on the tassel [10–13]. BR seems to play a prominent role in branching in monocots [13]. Components of the BR signaling pathway, including BZR1, are conserved in higher plants, including rice and maize [11, 13–15]. Suppressing the OsBZR family by RNAi leads to BR-insensitive phenotypes in rice and expression of dominant OsBZR1S156G-GFP rescued *Arabidopsis* *bri1* mutant, demonstrating the conserved function in monocots [16]. Genetic analysis has linked variations related to ZmBES1/BZR1-5 to kernel size, and overexpression of maize BZR1 homologs in *Arabidopsis* increased organ size [11, 14, 15]. The nuclear accumulation of maize BZR1 homolog ZmBES1/ZmBZR1 (hereafter simplified as ZmBZR1) is increased by BR treatment and decreased by RNAi knockdown of the BR receptor ZmBRI1 [17]. Such BR-dependent nuclear localization is similar to that observed for BZR1 in *Arabidopsis*, rice, and tomato [16, 18, 19]. These results indicate that ZmBZR1 plays a conserved role as the BR signaling transcription factor in maize. Here, to characterize the BR-regulated transcriptional network in maize, we identified genome-wide targets of ZmBZR1 via chromatin immunoprecipitation followed by high-throughput sequencing (ChIP-seq). Furthermore, we analyzed the genome-wide variation of ZmBZR1 binding using an internally controlled, quantitative ChIP-seq approach that we named hybrid allele-specific ChIP-seq (HASCh-seq), where allele-specific TF binding is quantitatively analyzed in F_1_ hybrids.

## Results

### Conservation and evolution among the transcriptional targets of BR signaling

**Fig. 1:**
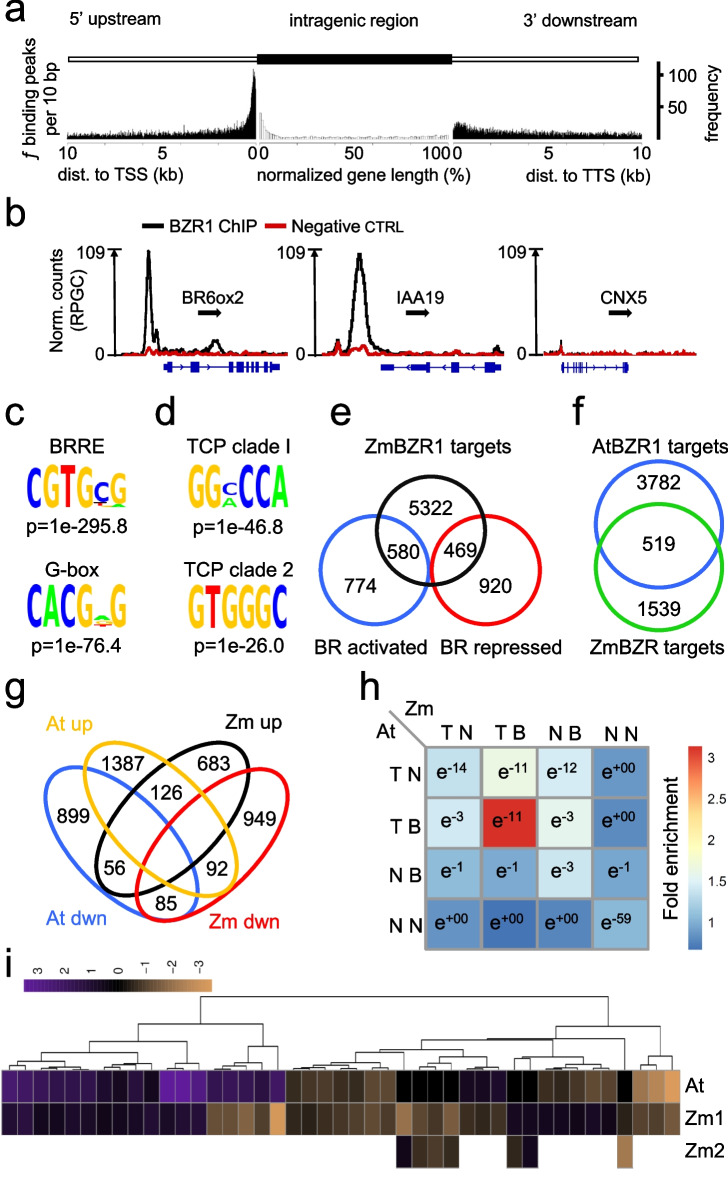
BZR1 regulatory network in maize and *Arabidopsis*. **a** Distribution of ZmBZR1 binding around transcribed genes. Frequency of ZmBZR1 binding peaks up to 10 kb up- or downstream of TSS or TTS and intra-genic, respectively. **b** ChIP-seq identified ZmBZR1 binding in proximity of putative targets repressed (*BR6ox2/BRD1*), induced (*IAA19*) or not controlled by BR (*CNX5*). Black line shows normalized BZR1 ChIP reads (reads per genome coverage, RPGC) and red line depicts the negative control (non-transgenic siblings). Genes are depicted in blue; black arrows indicate direction of transcription. **c**, **d** Significantly overrepresented ZmBZR1 binding motifs, BRRE (CGTG[C/T]G) and G-box (CACG[A/T]G) (**c**) as well as BRRE significantly co-localizing secondary motifs for TCP TF class I (GG[A/C]CCA) and class II (GTGGGC) (**d**) determined by GEM. **e** Direct and indirect targets of ZmBZR1. Shown is the overlap of BZR1 ChIP-seq and RNA-seq of the BR-deficient *brd1* mutant +/− BR. **f** Conservation of the BZR1 targets between *Arabidopsis* and maize (*Arabidopsis* orthologs). **g** Conservation of *Arabidopsis* and maize (*Arabidopsis* orthologs) BR up- and downregulated genes. **h** Overlap of orthologous BR-responsive (B) and non-responsive (N) BZR1 target genes (T) and non-target genes (N) in *Arabidopsis* (At) and Maize (Zm). Color-coding indicates fold enrichment compared to random expectation (blue, low enrichment to red, high enrichment). e^Numbers^ indicate *p*-values of significance of this enrichment assuming a hypergeometric distribution. **i** Heatmap of direct orthologues target genes of BZR1 in *Arabidopsis* and maize induced (green) or repressed (red) by BR. Overall, 65% of 1:1 orthologs and 72% of 1:2 copy orthologs of direct BZR1 targets between *Arabidopsis* and Maize, respectively, showed the same direction of BR regulation

To characterize the BR-regulated transcriptional network in maize, we performed RNA-seq on shoot tissue of wild-type as well as BR biosynthesis-deficient *brd1* (BR6ox2) seedlings treated with and without brassinolide (BL), the most active BR. We identified a total of 2743 BR-responsive genes, including 1354 BR-induced and 1389 BR-repressed genes (Additional file 1: Table S1). To identify the genes directly regulated by the BR signaling pathway in vivo, we performed ChIP-seq analysis of the maize BES1/BZR1 homolog (Zm00001eb325550_P002, Zm00001d021927), using the transgenic plants expressing a ZmBES1/BZR1-YFP fusion protein driven by the ZmBES1/BZR1 promoter described previously [17]. We backcrossed ZmBES1/BZR1-YFP six times with the B73 inbred line, which is among the most well-studied and annotated maize lines [20]. We confirmed that the nuclear localization of ZmBES1/BZR1-YFP responds to the BR biosynthesis inhibitor propiconazole (PPZ) [21, 22] and BR treatments (Additional file 2: Fig. S1). We performed ChIP-seq experiments using ZmBES1/BZR1-YFP with non-transgenic B73 as the negative control. Our ChIP-seq experiment identified 17,463 high-confidence ZmBZR1 binding peaks (Additional file 3: Table S2), most of which were near the transcription start site (Fig. 1a), and about 65% (11,232) overlapped with previously identified open chromatin regions [23]. The ZmBZR1 ChIP-seq peaks located near 6371 genes (Additional file 4: Table S3). The list of ZmBZR1 target genes included maize homologs of known AtBZR1 targets, such as *ZmBR6ox2* and *ZmIAA19* (Fig. 1b) [9], some of which were validated by ChIP-qPCR (Additional file 2: Fig. S1). Our analysis of *cis*-element enrichment identified the BR response element (BRRE, CGTG(C/T)G) and the G-box (CACGTG), which were previously identified as BZR1 binding sites in *Arabidopsis* [9, 24] (Fig. 1c). Interestingly, co-enrichment of the binding sites for TCP factors (e.g., GG^C^/_A_CCA) was also observed, similar to the observation in *Arabidopsis* [9] (Fig. 1d). Together, these results indicate that the BR response *cis*-elements and their combination with other factors are conserved in *Arabidopsis* and maize.

The ZmBZR1 binding targets include 38.5% of the BR-responsive genes, including 580 and 469 BR-activated and -repressed genes, respectively, identified in our RNA-seq analysis (Fig. 1e)*.* Overall, maize BZR1 target genes had 2058 unique *Arabidopsis* annotated orthologs, of which 519 (25.2%) were previously identified as AtBZR1 targets [25] (Fig. 1f, Additional file 5: Table S4, Additional file 2: Fig. S1). A smaller overlap was observed for the BR-responsive genes (Fig. 1g). We then classified orthologous genes between both species into BZR1 target only, BR responsive only, both or none of those two. Among the different groups, BR-responsive BZR1 targets showed the highest overlap (~3 fold higher than random chance) between the two species, with significant overlap also with non-responsive targets and BR-responsive non-target genes (Fig. 1h). Furthermore, the conserved BR-responsive target genes tended to show the same direction of response (Fig. 1i). The results indicate both conservation and divergence of the transcriptional targets of BR signaling.

To understand whether the species-specific targets represent functional divergence between Maize and *Arabidopsis* BZR1 signaling, we further performed a gene ontology analysis comparing functional enrichment of “*Arabidopsis*-only” (3782, [25]) and “maize-only” BZR1 (1539) targets (Additional file 6: Table S5). Only two terms were significantly enriched in *Arabidopsis* but not in maize. The enrichment of those two terms was, however, similar in maize but failed the significance test. On the other hand, twenty-one terms were maize exclusive, including multiple terms related to stress and stimuli responses such as response to biotic stimuli (Additional file 6: Table S5). Fourteen of those twenty-one terms, and both *Arabidopsis*-only terms, however, were also significantly enriched in the shared targets between *Arabidopsis* and maize BZR1. The results suggest that the biological functions of BR regulation are relatively conserved, while the specific target genes involved in the biological functions have diverged between *Arabidopsis* and maize. However, GO enrichment analysis has limited sensitivity and the large number of variations makes it very difficult to correlate specific target differences with phenotype differences between maize and *Arabidopsis*. We therefore focused on analyzing the intraspecies variation of the ZmBZR1 network.

### Hybrid allele-specific chromatin binding sequencing (HASCh-seq) identifies allele-specific binding of BZR1

**Fig. 2:**
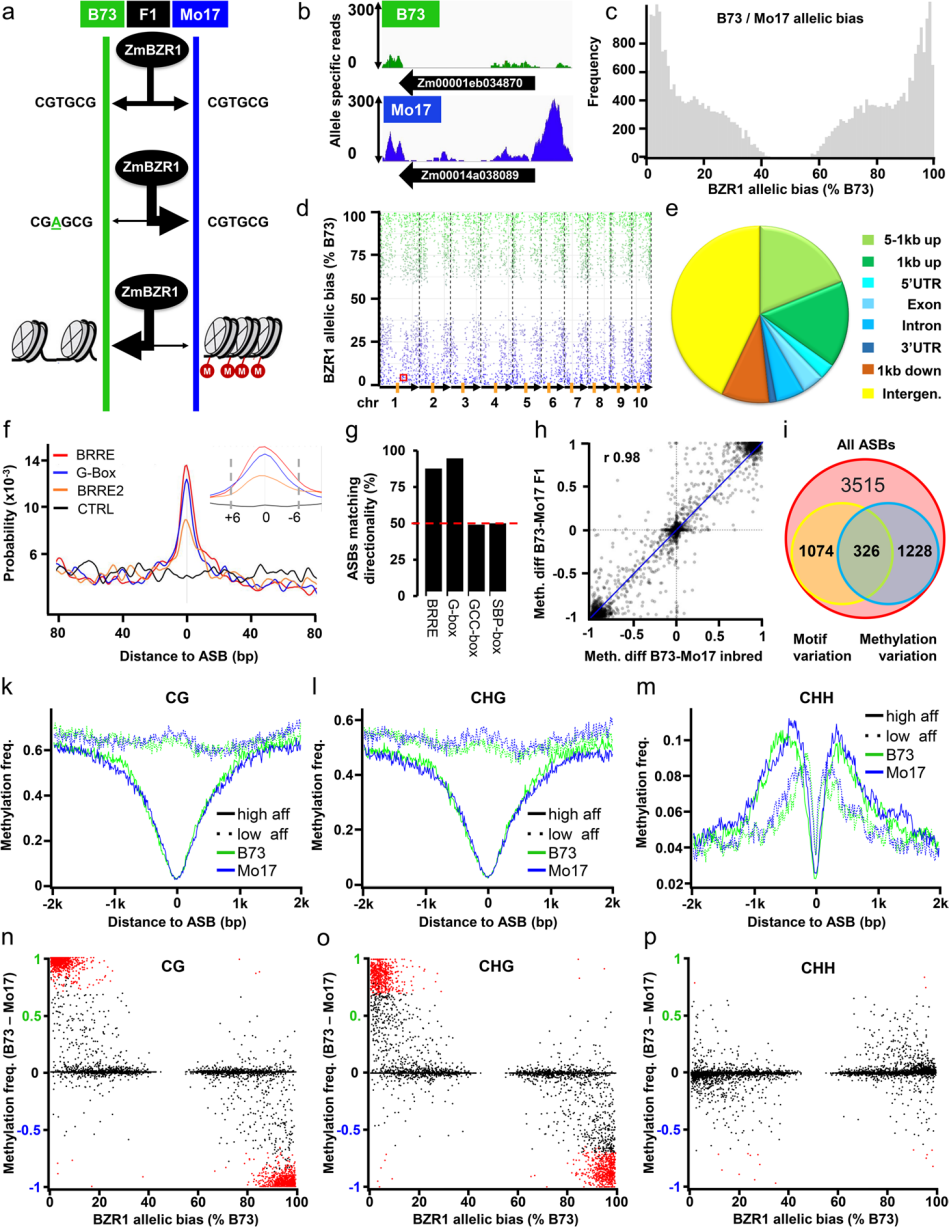
DNA sequence and methylation variation correlate with differential BZR1 binding. **a** Schematics of HASCh-seq approach and possible causes for allele-specific binding events. Chromatin-IP is performed in F_1_ hybrid plants. Possible scenarios for TF binding to the parental genomes (green and blue) are depicted. Binding strength is depicted by the black arrows width: (top) with no alteration in motif or chromatin structure, binding is expected to be equal. Lower binding is expected if the motif is altered (middle) or epigenetics like DNA methylation (bottom) vary between alleles. **b** Example of allele-specific ZmBZR1 binding near Zm00001eb034870. ZmBZR1 bound reads that map uniquely to B73 (green) or Mo17 (blue) are shown. **c** Distribution of SNPs with a significant allelic bias to either B73 or Mo17 located within BZR1 peaks. **d** Allelic and spatial distribution of ASBs with a bias towards B73 (green) or Mo17 (blue) along the B73 chromosomes. Allelic bias is expressed as a percentage of B73 read counts. Chromosome borders and length are depicted by dashed lines and arrows, respectively. Centromeres are indicated by orange rectangles. A red box highlights the ASB near Zm00001eb034870 displayed in **b**. **e** Genomic distribution of ASBs classified according to their location relative to genes. In case of two genes in the proximity of an ASB, the priority given was exon>intron>UTR>1 kb upstream>1 kb downstream>1–5 kb upstream. **e** Frequency of BRREs (CGTG[C/T]G), G-box (CACGTG), and a control motif CCGTAC (SBP-box) around ASBs of the alleles with higher BZR1 binding. **g** Fraction of ASBs overlapping with motifs, for which the allele with canonical BRRE, G-box or control motifs GCCGCC (GCC-box), and SBP-box showed higher ZmBZR1 affinity. Both BZR1-related motifs, but not the control motifs, diverge significantly (*p*<0.001, Fisher’s exact test) from the expected 50% random distribution. **h** Correlation of haplotype-specific DNA methylation differences at ASB loci between B73/Mo17 parental alleles and B73/Mo17 F_1_ alleles (*r* Pearson correlation). **i** ASBs affecting ZmBZR1 binding motifs and/or overlapping with allele-specific methylation differences (CpG, CHG, or CHH) in the F_1_. **k–m** Average **k** CpG, **l** CHG, and **m** CHH methylation frequency in B73 (green) and Mo17 (blue) over ASB loci with a least 85% binding bias towards B73 or Mo17. High-affinity (^**___**^) and low-affinity (^**….**^) alleles are separated by genotype. **n–p** Correlation of CG (**n**), CHG (**o**), and CHH (**p**) methylation with allele-specific ZmBZR1 binding. Average B73-Mo17 methylation of the 20-bp surrounding ASBs are plotted against the allelic bias (expressed in percentage of B73 read counts). Significant methylation differences are indicated by red dots

To understand the functions of BZR1 binding in regulating gene expression and plant traits, we studied the influence of genetic variation between two inbred lines, B73 and Mo17, on BZR1 binding. ChIP-seq is considered technically challenging particularly for quantitative comparison. To minimize biological and technical variations between ChIP-seq experiments, we decided to perform ChIP-seq in F_1_ hybrid plants. Genetic variations that affect BZR1 binding will show a shift of the allele frequency after ChIP from the expected 1:1 ratio in an F_1_ (Fig. 2a, b). We named this strategy of ChIP-seq in F_1_ HASCh-seq (hybrid allele-specific chromatin binding analysis). We chose B73 and Mo17 as they are among the most diverse maize inbred lines and their hybrid is one of the most studied [26–28]. They also show differences in many phenotypes that are affected in BR mutant maize, such as plant height, tassel branching, flowering time, leaf width, and leaf angle [26, 28]. The DNA-binding domain of BZR1 in B73 and Mo17 has an identical sequence (Additional file 2: Fig. S2), and therefore the variation of the BR response gene network should be due to variation of target DNA. To identify the genome-wide variations in BZR1 binding, we performed six independent crosses (3x ZmBES1/BZR1-YFP/B73xMo17 and 3x Mo17xZmBES1/BZR1-YFP/B73) and performed six replicates of HASCh-seq experiments. The reads were mapped to a concatenated B73 and Mo17 genome, and uniquely mapping reads were used to define BZR1 binding sites and to quantify the relative haplotype binding (Fig. 2b). The results showed a strong reproducibility between the biological replicates (Pearson’s correlation coefficient > 0.88, Additional file 2: Fig. S1). Combined analysis of the ChIP-seq results identified 52,765 high-confidence BZR1 binding peaks (Additional file 7: Table S6) flanking 13,208 genes (Additional file 8: Table S7) in the hybrid. When analyzing the allelic ratio at all heterozygous SNPs located within high-confidence BZR1-binding peaks, a total of 33,267 SNPs (Additional file 9: Table S8) showed a significant allelic bias (adjusted *p* < 0.001), without an overall bias to either genome (Fig. 2c). These SNPs were divided into 7817 independent linkage groups. For the downstream analysis, the lead SNP of each linkage group (i.e., closest to the binding summit) was considered as putative allele-specific BZR1 binding site (ASB). Finally, we excluded ASBs that showed a significant bias (*p*<0.05) in their surrounding region in the ChIP-input data (i.e., before immunoprecipitation, Additional file 2: Fig. S3), to avoid potential artifacts (e.g., mapping artifacts or errors in whole genome alignment) resulting in 6143 ASBs (Fig. 2d, Additional file 10: Table S9).

About 57.1% of ASBs were found within 5 kb upstream to 1 kb downstream of 2424 genes, with 15.9% of ASBs locating within 1 kb upstream of the TSS, which is about 1.5% of the genome (Fig. 2e). The 2424 flanking genes were considered putative BZR1 ASB target genes (Fig. 2e, Additional file 11: Table S10). We hypothesized that ASBs outside genic regions may be located in intergenic enhancers. We thus analyzed their abundance, compared to background SNPs (bgSNPs, see “Methods”) with the same average genomic distribution, in the 1495 intergenic enhancer regions identified in B73 previously [29]. We found 7.8% (213/2730) of the non-genic ASBs coincided with the intergenic enhancer regions [29], which is approximately 85-fold higher than for bgSNPs (125/136,500 non-genic bgSNPs). In addition, there was a much higher density of ASBs than bgSNPs surrounding the enhancer regions (Additional file 2: Fig. S4).

An analysis of the higher affinity allele sequence of ASB regions revealed the BZR1-binding motifs (BRRE and G-box) as the most enriched (Fig. 2f). This enrichment dropped off within 6 bp around ASBs, indicating that allele-specific BZR1 binding was directly overlapping with variants in BRRE/G-box motifs (Fig. 2f). Of the ASBs that overlapped with BZR1-binding motifs (i.e., were altered compared to the canonical motif), decreased BZR1 binding was observed for 87.7% and 94.7% of variations in BRRE and of G-box motifs, respectively. In contrast, no such canonical bias was found for the TF motifs GCC-box (49.1%) and SBP-box (50.0%) (Fig. 2g). In total, we identified 1400 variations in BZR1 binding motifs, which accounted for 23% of all ASBs (Additional file 12: Table S11). The ASBs not associated with variation of BZR1 binding motifs could be caused by variation in binding motifs for BZR1-interacting TFs, such as auxin-response factors (ARFs) and phytochrome-interacting factors (PIFs) [25, 30, 31] or haplotype-specific DNA methylation which affects TF binding [27, 32, 33].

### High-affinity BRZ1 binding alleles are largely hypomethylated

To determine whether haplotype-specific DNA methylation correlates with ASBs, we performed enzymatic Methyl-seq of B73xMo17 hybrids and identified haplotype-specific DNA methylation. We compared our results with the methylation data from the inbred lines B73 and Mo17 [27] and found consistent variations (Pearson correlation 0.98) in haplotype-specific DNA methylation at ASB loci in F_1_ and parental lines (Fig. 2h). These results are consistent with previous observations of a mostly persistent methylation status between inbred and F_1_ generations [27, 32]. We found significant (following [27], one allele ≤10% methylated and the other ≥70%) haplotype-specific DNA methylation for 25.3% (1554) of ASBs, including 5.3% (326) ASBs that also overlapped with variation in BZR1 motifs (Fig. 2i, Additional file 12: Table S11). About 99% of the haplotype-specific DNA methylation events at ASBs were CG or CHG, and only 1% were CHH methylation. There were strong correlations between reduced BZR1 binding and hypermethylation in both CG and CHG contexts, which are known to be associated with repression of transcription [27] (Fig. 2k,l). In contrast, CHH methylation accumulated in the regions flanking BZR1 binding (Fig. 2m). Unlike CG and CHG methylation, CHH methylation is known to be associated with expressed genes [33]. For the majority of ASBs (66.6%, 4092/6143), both alleles were not methylated (<10% methylated Cs around ASBs). Among the differentially methylated ASB loci, 99.6% (1536/1542) showed BZR1 binding bias towards the hypomethylated CG or CHG alleles (Fig. 2n–p). These results indicate that differential CG and CHG methylation strongly correlate with a major portion of ASBs.

### Allele-specific BZR1 binding is correlated with allele-specific expression

**Fig. 3:**
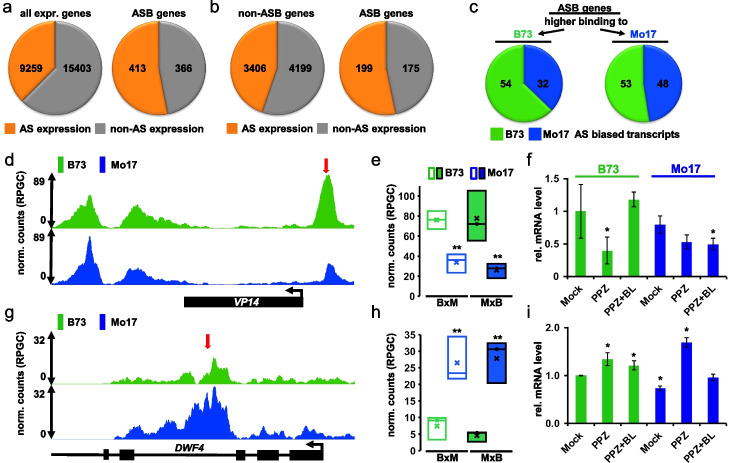
ASBs correlate with allele-specific mRNA abundance. **a**, **b** Fraction of (left panel) all expressed (> 20 reads) and orthologous maize genes or (right panel) genes with an ASB in their 3 kb promoter displaying allele-specific (AS) mRNA abundance differences (orange) between **a** B73 and Mo17 parental alleles [34] and **b** B73 and Mo17 alleles in F_1_ hybrids. **c** Fraction of ASB genes with higher BZR1 binding to B73 (left) or Mo17 (right) and the mRNA levels of the nearby gene being biased to B73 (green) or Mo17 (blue). **d**, **g** Allele-specific, cumulative HASCh-seq signal around ASBs near *VP14* (**d**) and *DWF4* (**g**). Normalized HASCh-seq reads (reads per genome coverage) mapping uniquely to B73 (green) or Mo17 (blue) are shown. **e**, **h** Box plots of allelic reads at two selected ASBs (red arrows in panels d and g, respectively) from the three replicates for B73xMo17 (BxM) and Mo17xB73 (MxB) F_1_ hybrids. **p<0.01 **f**, **i** Transcript levels of the BR-induced *VP14* (**d**) and BR-repressed *DWF4* (**g**) measured by qRT-PCR in B73 and Mo17 plants treated with mock, BL inhibitor PPZ, or PPZ plus BL for 4h. *p<0.05

To determine whether variations of BZR1 binding contribute to differential gene expression, we compared our ASB data with the transcript levels of B73 and Mo17 alleles in both parents and B73xMo17 hybrid lines. About 37.5% (9259 of 24,662) of all expressed genes with orthologs in both inbred lines showed allele-specific mRNA differences between B73 and Mo17 plants (Fig. 3a) [34]. A higher portion (53.0%, 413 of 779) of genes with an ASB in their promoter showed allelic variation in mRNA between B73 and Mo17 plants (*p*=0.0001, Fig. 3a). We also performed RNA-seq of B73xMo17 hybrids. The experiment quantified allele-specific expression of 7605 genes including 374 genes with an ASB in their promoters. We found that 199 (53.2%) of those ASB genes showed allele-specific transcript differences, which is a significantly (*p*=0.0009) higher portion than genes without an ASB in their promoter (44.4%, 3207/7231) (Fig. 3b, Additional file 13: Table S12). There was no obvious correlation between BZR1 allele-specific binding and the direction of change in expression level (Fig. 3c), which is expected given that BZR1 acts as an activating or repressing TF for different target genes (Fig. 1d) [25]. However, RT-qPCR analysis of individual ASB-associated genes, in B73 and Mo17 plants, showed the expected correlation of BZR1 binding with increased expression of the BR-induced gene *VP14* [35] (Fig. 3d–f), and decreased expression of the BR-repressed gene *DWF4* [36] (Fig. 3g,h). Sequence analysis revealed that the ASB in the *DWF4* intron overlapped with a BRRE motif in Mo17 (CGTGTG) which was altered in the B73 allele (TGTGTG). This is consistent with the weaker BZR1 binding (B73 6.6, Mo17 27.7 normalized counts) and lower BR/PPZ response (Fig. 3g–i). Similarly, the ASB in the *VP14* promoter overlapped with the core (TGTC) of an ARF binding motif, which was altered in Mo17. ARF TFs have been shown to directly interact with BZR1 [25], and indeed, we found a BZR1 G-box element (CACGTG) only 9 bp upstream which, unlike the ARF element, was shared between B73 and Mo17. A possible explanation for the two thirds lower BZR1 binding to the Mo17 *VP14* promoter allele could be the reduced binding of an ARF/BZR1 heterodimer. Taken together, these results suggest that variation of BZR1 binding contributes to variation of gene expression.

### ASBs are linked to trait diversity in maize

**Fig. 4:**
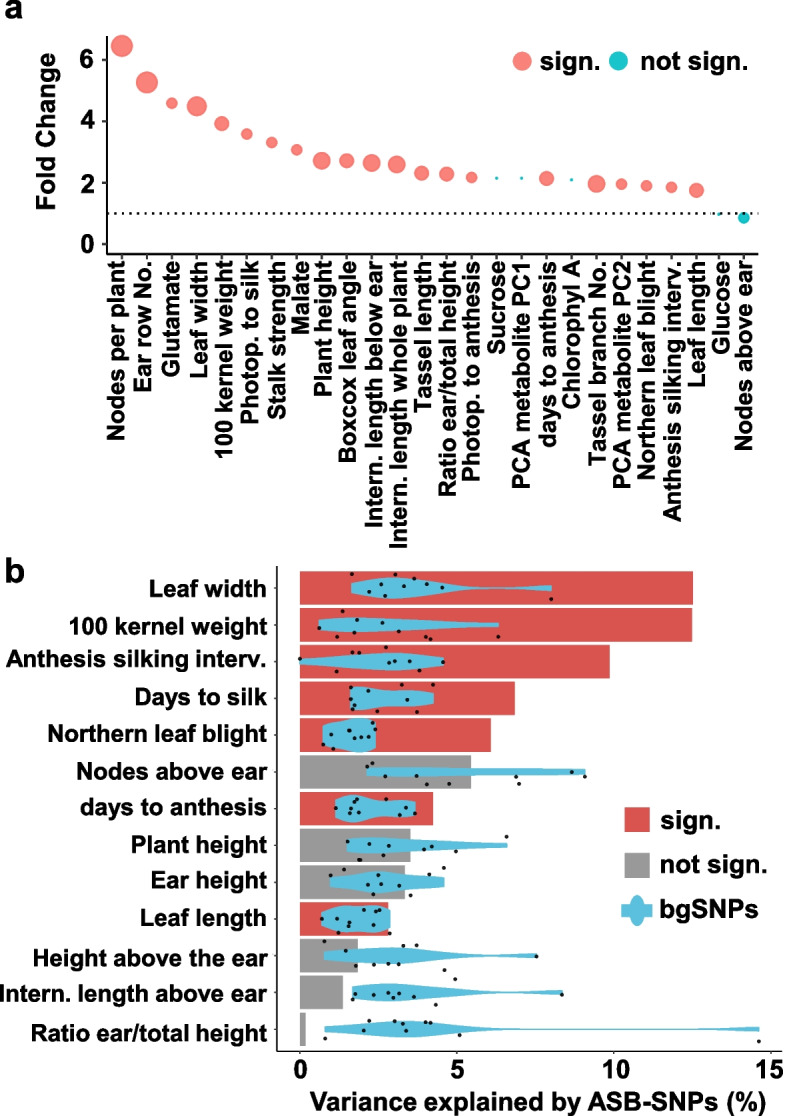
ASBs are linked to growth and disease-related traits. **a** Association of ASBs with nearby (+/− 2 kb) 4015 significant GWAS hits curated by [2] for selected phenotypes. Abbreviations: Intern.: internode; Interv.: Interval; No.: Number; Photop. Photoperiod. **b** VCAP Variance component analysis. Variance explained (h2) by the ASB SNP set (bars) and background SNP set (violin plots, derived from permutation results). Red color bars denote a significantly higher variance explained by ASBs than expected by chance (one-sided permutation test < 0.1)

To assess the relationship between variations of BZR1 binding and trait variations, we quantified the enrichment of 4015 GWAS hits across 41 traits [2] within 2 kb of ASB regions. We found a 1.8-6.5 fold and significant enrichment (*p*<0.05) for 21 of the traits, compared to the bgSNPs with the same average genomic distribution and allele frequency. The largest fraction (52.4%, 2-fold enrichment within the dataset) were traits related to growth and yield, as expected based on the main functions of BR known from extensive studies in *Arabidopsis*. Interestingly, other known phenotypic variations between B73 and Mo17, such as tassel branching [34] (13 associated ASBs) and disease resistance [37] (11 associated ASBs), were also enriched (Fig. 4a and Additional file 14: Table S13). To further investigate the role of ASBs in complex organismal trait variation, we used variance component annotation (VCAP), which partitions the heritable phenotypic variance into annotation-specific components classifying ASB and bgSNP regions [5]. We examined the maize Nested Association Mapping population (NAM), which captures a remarkable degree of genetic diversity in a relatively small panel with 25 founder lines. The NAM design simultaneously exploits the advantages of both linkage analysis and association mapping [38]. We used the NAM for the VCAP of ASBs and found that they explained a remarkable portion of the heritable variance (> 10%) for some of the traits with moderate to high heritability (h2 > 0.4l). For 7 of the 13 traits analyzed, we found that ASBs explained disproportionately larger genetic variances compared to the bgSNPs, including leaf width, 100 kernel weight, and northern leaf blight (NLB) resistance, but not nodes above the ear or the ratio of ear height to total height (Fig. 4b).

**Fig. 5:**
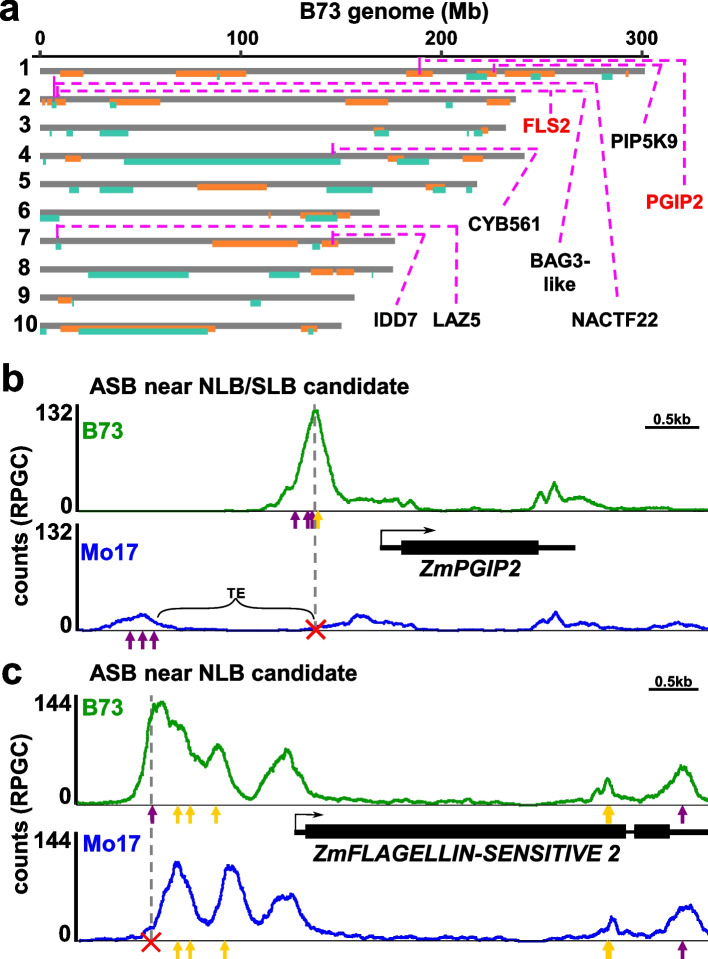
ASB candidate genes near disease-associated GWAS hits. **a** Genome-wide joint linkage map (chr1-10 top to bottom) of NLB (orange) and SLB (turquoise) disease QTLs (NAM population) and ASB candidate genes (pink lines) that co-localized with NLB or SLB GWAS hits are highlighted. *IDD7* (Zm00001eb320600), *LAZ5* (Zm00001eb304160), *CYB561* (Zm00001eb185950), *BAG3*-like (Zm00001eb070420), *NACTF22* (Zm00001eb070490), *PIP5K9* (Zm00001eb044280). **b**,**c** Allele-specific, cumulative HASCh-seq B73 (green) or Mo17 (blue) signal (reads per genome coverage) around ASBs that co-localized with GWAS hits for northern and southern leaf blight near **b** *ZmPGIP2* (Zm00001eb034870) and **c** *ZmFLS2* (Zm00001eb070510). Yellow and purple arrows depict BRRE and G-box motifs underneath peaks, respectively. Red cross highlights altered motifs. Gray dashed connected lines depict ASB positions aligned to B73 and Mo17

To further explore potential links between ASBs and disease traits, we identified 11 ASBs which co-localized (within 2 kb) with NLB/SLB GWAS hits [2]. We found that these ASBs, and their nearby genes, also coincide with joint linkage mapping QTLs for NLB and SLB in the NAM population (Fig. 5a). As an example, a significant difference in BZR1 occupancy upstream of the TSS of a gene with high homology to polygalacturonase-inhibiting proteins, *PGIP2* (Zm00001eb034870), was observed (Fig. 5b). PGIPs are cell wall proteins that inhibit the pectin-depolymerizing activity of polygalacturonases secreted by microbial pathogens and insects [39]. *ZmPGIP2* is a candidate gene for both northern and southern leaf blight resistance [37, 39, 40] and is upregulated by rice black-streaked dwarf virus which causes maize rough dwarf disease [41]. BZR1 binding was significantly higher (6.6-fold) for the B73 allele compared to Mo17 (Fig. 5b). The B73 peak allele included a BRRE and three G-box-like (2x CACGTG and CACGTT) motifs, whereas the Mo17 allele had a SNP in the BRRE and a HIP-superfamily helitron insertion between the BRRE and G-box motifs. This shifts the Mo17 G-box elements 1.5 kb upstream, where only a small BZR1 peak was detected (Fig. 5b). Closer inspection showed that the B73 peak allele was hypomethylated, whereas the 1.5 kb upstream Mo17 peak was hypermethylated. The transcript ratio of B73 to Mo17 *ZmPGIP2* alleles was 1.2 to 1.61 (*p*<0.05, [34]) and 1.6 (*p*=0.055, in inbred and F_1_ lines based on RNA-seq data, respectively (Additional file 13: Table S12). Similarly, the closest maize homolog of *Arabidopsis* pathogen-associated molecular patterns (PAMP) flagellin receptor FLAGELLIN SENSITIVE 2 (ZmFLS2, Zm00001eb070510) [42] located near SNPs associated with NLB [2] and maize stalk rot [43]. Upon flagellin perception, AtFLS2 rapidly forms a complex with the BRASSINOSTEROID INSENSITIVE1 ASSOCIATED KINASE1 (BAK1) co-receptor [44, 45]. *ZmFLS2* expression is affected by *Pseudomonas syringae* (bacterial brown spot), *Fusarium graminearum* (stalk rot) [43], and in a *Puccinia polysora* (southern corn rust) tolerant line [46]. B73 and Mo17 alleles of *ZmFLS2* showed multiple BZR1 binding sites in the promoter (Fig. 5c). The largest binding peak in B73, however, was greatly reduced in Mo17. This B73 peak summit contained a G-box motif that was altered in Mo17 (Fig. 5c). The mRNA ratio of B73 to Mo17 *ZmFLS2* alleles was 1.38 (*p*<0.05, [34]) and 1.91 (*p*<0.05) in inbred and F_1_ lines, respectively (Additional file 13: Table S12). Considering the differences observed between B73, Mo17, and other NAM founders in their resistance to NLB and SLB [37, 39, 40], these ASBs linked to GWAS hits provide strong candidates for future functional studies.

## Discussion

Hormones have major effects on plant growth and development. Genetic variations in plant hormone pathways have great potential for improving crop yield. For example, genetic variations in hormone synthesis and signaling pathways contributed to the green revolution [47]. As a major growth-promoting hormone, BR controls important agronomic traits, such as plant height and architecture, branching, flowering time, fertility, seed size, and disease resistance [10, 12]. Although the hormone networks have been studied extensively at the molecular level, genetic variations within these networks and their contributions to trait variations remain poorly understood. Our study provides the first comprehensive genome landscape of BR targets and their variations between two maize haplotypes. Our results show a high degree of variation in the BR target genes and linkage between hundreds of BZR1 binding variants to QTLs for important traits.

Our results suggest that BR, through ZmBZR1, is involved in the regulation of thousands of target genes in maize, analogous to findings in *Arabidopsis* [48]. Previous studies found that the BR signaling pathway seems to be conserved in higher plants [49], raising the question of how such a conserved signaling pathway contributes to phenotypic variations. Our results suggest that the BZR1-binding sequence specificity is conserved in maize, but the distribution of the *cis*-elements in the genome and thus the target genes of BZR1 is highly variable between maize and *Arabidopsis* and even between two maize inbred lines.

Our finding of large numbers of ASBs is consistent with the notion that the *trans*-*cis* interface of signaling pathways, constituting numerous binding sites of TFs, is a major target of evolution to fine-tune or reshape cellular regulatory pathways. Nearly half of quantitative trait variations in maize are explained by accessible, non-coding regions that may contain regulatory elements, e.g., TF binding sites [2, 5]. Pinpointing the causal variations in non-coding sequences is challenging because (1) the sequence does not reliably inform function (unlike coding variations), (2) there is a higher densities of SNPs in non-coding than coding regions, and (3) the effect size of individual non-coding variation tends to be small [50]. However, collectively, a large number variation in the binding sites of a transcription factor may reshape or modify the outputs of a signaling pathway [50]. Our study demonstrates that large numbers of variations in non-coding regulatory sequences affect ZmBZR1 binding, potentially causing quantitative changes in gene expression and phenotypes.

While promoters are defined by their proximity to genes, distant enhancer regions, although important for gene regulation, are more difficult to pinpoint, in particular in plants, as they frequently lack the specific marks often associated with enhancers in animals [23]. Oka et al. recently identified over a thousand such putative enhancers in B73 using various histone marks, chromatin accessibility, and DNA methylation [29]. The finding of ASBs enriched in those enhancer regions provides evidence for BZR1 functions through enhancers, which was not known previously.

While molecular approaches such as traditional ChIP-seq have long been used to identify TF binding sites, quantitative analyses of the effects of genetic and epigenetic variations on TF binding often suffer from sample-to-sample variations. Furthermore, when TF-DNA binding is analyzed in separate genetic samples, the contributions of variation in TF abundance and *cis*-elements cannot be distinguished. By performing HASCh-seq analysis of allele-specific BZR1 binding in F_1_ hybrid plants, we avoided both sample-to-sample variation and *trans* effects. Another approach is DNA Affinity Purification and sequencing (DAP-seq), which analyzes in vitro TF binding to genomic sequences. While DAP-seq analysis can also identify variations in DNA sequence or methylation that affect TF-DNA binding, many in vivo binding events involve interaction between TFs or heterodimerization of TFs and are not identified by DAP-seq using a single TF. The DAP-seq analysis of *Arabidopsis* BZR1 identified only about 8.5% of the targets identified by ChIP-seq [25, 51]. Indeed, we found variations in BZR1-binding sequence and DNA methylation correlated with only about half of ASBs. The remaining ASBs could be due to variation in binding sites of BZR1’s partners, such as ARFs and PIFs [25, 30]. Such in vivo ASBs mediated by putative partners would be missed in traditional DAP-seq, but can potentially be tested in future DAP-seq experiments using BZR1 in the presence of its partners.

ASBs identified in the F_1_ reflect the variations of DNA sequence and methylation between the two haplotypes. The effect of a motif variation on BZR1 binding is likely similar in the F_1_ and parental lines, as the DNA-binding domain of ZmBZR1 has an identical sequence in B73 and Mo17. However, other trans-acting factors may not be identical in F_1_ and either parent, and thus the impact on gene expression and phenotype could be confounded by other factors that are different in the F_1_ and the two parents. This is especially expected in plants which show strong heterosis, such as maize. Similarly, phenotype data used for GWAS predominantly comes from inbred lines. While the DNA sequence and most DNA methylation status are maintained in the F_1_, some epigenetic differences such as chromatin status between parental lines might be lost in the F_1_ and thus missed by HASCh-seq but detected in the parental line.

We identified 1400 variations of canonical BZR1-binding sites, of which ~90% were associated with a decreased BZR1 occupancy. These represent only 23% of all the cases of differential ZmBZR1 occupancy. However, this is a stringent dataset restricted to the lead SNPs of ChIP-seq peaks. There may be additional variants in BZR1 binding sites if all biased SNPs in ChIP-seq peaks were included. Another possible explanation is that DNA methylation, which in vitro has been shown to reduce the interaction of most TFs, including *Arabidopsis* BES1/BZR2, with their target sequence [51], may contribute to variation in ZmBZR1 binding in vivo. Indeed, at about 25% of the ASBs, allele-specific DNA methylation correlated strictly with decreased BZR1 binding. While TFs can also influence DNA methylation, as recently described in animal systems [52], the reduced BZR1 binding was correlated with CG and CHG methylation, which is largely stable between generations [27]. Consistent with this, CG and CHG methylation at ASB loci was stable between inbred and F_1_ lines as well as with or without exogenous BR treatment. Our results thus suggest that the DNA methylation contributes to variation in ZmBZR1 binding in vivo. The remaining ASBs that displayed neither changes in BZR1 binding motifs nor in DNA methylation may be due to variation in non-canonical BZR1 binding sites, binding sites of TFs that interact/recruit BZR1, or other epigenetic mechanisms such as histone modifications. Our results are consistent with an allele-specific TF binding analysis in human cell cultures, which also found only a minority of putative causative variants in the canonical motif [8].

While phenotypic impact of each ASB could be further evaluated, e.g., by CRISPR mutagenesis, we believe integrating GWAS with ASB data is an effective way to correlate ASBs with phenotypic traits and select candidate ASBs for future functional tests. Co-localization of ASBs with GWAS hits and VCAP enrichment support the notion that variation of BRZ1 binding is linked to trait variation. Among the enriched traits, those related to growth and yield, including known BR functions (e.g., leaf width, leaf angle, plant height, and seed weight), were particularly prominent. In addition, our results hint at a potential role for ZmBZR1 in disease resistance. We also explored a potential role of BR in the NLB (caused by *Setosphaeria turcicum*). B73 and Mo17 plants were either treated with the BR inhibitor PPZ, or mock prior to inoculation with *S. turcicum* spores. Resistance to NLB was scored as incubation period, the number of days following inoculation when the first necrotic lesion appears. B73, but not Mo17, plants treated with the PPZ showed significantly longer incubation peroids (more resistant) to NLB than mock-treated plants (Additional file 2: Fig. S5). However, further experiments are needed to prove a role of BR and BZR1 in NLB resistance and rule out a BR-independent effect of PPZ. Lastly, we identified ASB genes located near multiple GWAS SNPs, which overlapped with complex differences in ZmBZR1 binding pattern, including sugar transport protein 4 (*ZmSTP4*, Zm00001eb324180, Additional file 2: Fig. S6).

## Conclusions

We present HASCh-seq as a robust method for identifying genetic variants that affect TF binding in plants. By analyzing the TF binding to two different alleles in the F_1_ hybrid, we avoided technical variations that compromise quantitation and trans-factor differences that complicate data interpretation. Our analysis of ZmBZR1 demonstrates a high level of variations in the BR transcriptional regulatory network between two diverse maize inbreds. A large portion of the differences of ZmBZR1 occupancy were correlated with variations in its binding motif sequences and DNA methylation status. Our data also provides genetic evidence for the functions of thousands of *cis*-elements in the BR transcription network in maize. The approach complements classical GWAS approaches, as there were significant associations between ASBs and BR-regulated traits. This demonstrates that combining GWAS with HASCh-seq can be a powerful approach to pinpoint candidate targets for genome editing to improve traits.

## Material and methods

### Plant material and growth conditions

Construction of the ZmBES1/BZR1-YFP transgenic line was previously described [17] and obtained in the HiII background from A.W. Silvester. B73 and Mo17 wild-type inbred seeds were obtained from the Germplasm Resources Information Network (GRIN). To backcrosse ZmBZR1-YFP from its original HiII (B73xA188) background, transgenic lines were used as pollen and B73 as ear donor to eliminate cross contamination. Six backcross lines were independently backcrossed six times, using heterozygous plants to avoid gene silencing. Lastly, B73-BC5 lines were used as pollen and ear donors for Mo17xB73BZR1-YFP and B73BZR1-YFPxMo17 crosses, respectively. For allele-specific analysis, tissues from 12 plants were pooled per replicate. The residual HiII regions cannot be completely removed but were minimized by the backcrosses, sample pooling and further addressed by deep sequencing of the input. Wild-type and ZmBES1/BZR1-YFP, and BR-deficient mutant (*brd1*) plants were grown side by side in greenhouses, under long-day conditions (16h day/8h night, 28–30°C), and in the 2013–2016 Carnegie Institution for Science summer fields (Stanford, California, USA).

### B73 ChIP-seq; B73 / Mo17 HASCh-seq, and ChIP-qPCR

ZmBZR1-YFP/B73, Mo17 inbred as well as ZmBZR1-YFP/B73xMo17 and Mo17xZmBZR1-YFP/B73 F_1_ hybrid plants and their non-YFP-carrying siblings (as negative control) were grown under greenhouse conditions for 26 days. The oldest 2 leaves were removed and 2 cm of meristem-enriched tissue was used (Additional file 2: Fig. S1). Per replicate *n*=12 plants were pooled. Tissues were first treated with 1 µM BL for 4 h at room temperature in water. After BR treatment, tissues were cross-linked with 2% formaldehyde for 10 min under vacuum with 5 min incubation after release. Tissues were homogenized to a fine powder in liquid nitrogen, and nuclei extraction was performed as described in [25]. Nuclear extracts were sonicated using a Branson 250 Sonifier (2× 4 min on time, 20 s on/off cycle with 10 min rest between repeats, 20 % amplitude), and after removing an input aliquot, incubated for 2 h with 10 µg polyclonal Anti-GFP antibody [25] (Additional file 2: Fig. S1). Protein-DNA complexes were captured on Dynabeads-Protein G (Life Technologies, #10003D), and the beads were washed with low-salt buffer (50 mM Tris-HCl at pH 8.0, 2 mM EDTA, 150 mM NaCl, 0.5% Triton X-100), with high-salt buffer (50 mM Tris-HCl at pH 8.0, 2 mM EDTA, 500 mM NaCl, 0.5% Triton X-100), with LiCl buffer (10 mM Tris-HCl at pH 8.0, 1 mM EDTA, 0.25 M LiCl, 0.5% NP-40, 0.5% deoxycholate) and twice with TE buffer (10 mM Tris-HCl at pH 8.0, 1 mM EDTA) and eluted with elution buffer (1% SDS, 0.1 M NaHCO3) at 65°C overnight. After a column purification (Quiagen, PCR purification kit), ChIP-seq libraries were generated using the Ultra II kit (NEB), following the manufacture’s recommendations using 10 ng per sample as starting material. ChIP-qPCR was performed using the Bioline SensiFAST SYBR Kit following the manufacturer’s recommendations on a Roche LightCylcer 480 at 63°C annealing temperature. Primers used for the analysis are listed in Additional file 15: Table S14 and F_1_-sequencing information in Additional file 16: Table S15.

### Enzymatic methyl-seq

Leaf tissue from BZR1-YFP/B73xMo17 F_1_s was harvested (*n*=6 plants, 3 replicates) and treated the same way (including BL treatment) as described for ChIP but without crosslinking. Tissues were homogenized in liquid nitrogen, and DNA was isolated with the DNeasy Plant Mini Kit (Qiagen). Libraries were prepared using the NEBNext Enzymatic Methyl-seq Kit (NEB) following the protocol for large DNA inserts. Therefore, 200ng genomic DNA was combined with 0.002 ng CpG methylated pUC19 DNA and 0.04 ng unmethylated lambda DNA. Fragmentation was done by using the Diagenode Bioruptor NGS in three rounds, 30s on, 90s off. Agilent Technologies 4200 Tape Station was used to determine the size distribution and concentration of the libraries.

### ChIP-seq data analysis

Quality-filtered ChIP-seq reads were aligned to the B73 AGPv4 genome using bwa-mem (v. 0.7.16a) [53] with default parameters, followed by removal of PCR duplicates using samtools (v. 1.3.1.) [54]. To determine BZR1 binding peaks, IP and negative control samples, after normalization for read depth, were analyzed using the GEM package (v. 3.0) [55] (using parameters: --fold 5, --k_min 5, --k_max 8). After samples were analyzed individually, peaks reproducible in all 3 replicas, using the GEM peak summits +/− 200 bp around, were determined using R (v. 3.3.2) and considered high-confidence peaks.

### HASCh-seq data analysis

To analyze the HASCh-seq data, we created a diploid genome concatenating the recently released B73 V5 genome with the Mo17 CAU genome [20, 56]. Potential adapter contamination and low-quality reads were removed using Seqpurge (v2019-03-26). Reads were then mapped to the B73xMo17 genome using STAR [57] (v.2.7.10a), with the options --alignIntronMax 1 to allow DNA mapping. Only uniquely mapping reads (MAPQ 255) were retained and duplicates removed with samtools (v1.9). Bam files were converted to normalized bedgraph and bigwig formats using bamCoverage (deeptools v3.5.1) with parameters --effectiveGenomeSize 3491781308 (determined using unique-kmers.py -q -k average readlength), --normalizeUsing RPGC, --exactScaling, --smoothLength 0, --binSize 1).

BZR1 binding peaks were determined using the GEM (v3.4) pipeline described using IP samples against the negative control obtained from ChIP on non-YFP sibling plants. First high-quality peaks were called using the merged file of all ChIP-seq replicas (parameters --k_min 6, --k_max 8, qval 0.001, 10:1 IP:control cutoff). To obtain enough coverage for GEM peak calling in the replicates, we combined the 6 into 3 replicates (1x B73xMo17 and 1x Mo17xB73 replicate) (parameters changed: qval 0.01, 5:1 IP:control cutoff). Only high-quality peaks that overlap with peaks in all 3 replicates were retained using bedtools (v2.29.0). Although our focus was to perform allele-specific analysis, we also include a peak file of the merged hybrid BZR1-YFP ChIP-seq data compared to the negative control in all replicates that contains not only unique, but also shared peaks between the B73 and Mo17 genome (Additional file 17: Table S16).

Whole genome alignment between B73 AGPv5 and Mo17 CAU was performed with progressive cactus [58]. SNPs between B73 and Mo17 and their respective matching coordinates were determined with the halSnps function of progressive cactus, using parameters “unique” and “noDupes”. At those SNP positions, reads were counted per allele using bedtools and awk.

ZmBZR1-YFP ASBs were determined using custom R (v. 3.3.2) scripts. In order to accurately access allele frequencies of all homozygous SNPs, we set a minimum read coverage cutoff of ≥ 1 reads for both alleles and ≥ 25 for at least one of the alleles, neglecting SNPs located on scaffolds (*n*=429,236). Of the remaining 429,236 SNPs, we determined significant variation of median allele frequency of 0.494 using a binomial test with a *p*-value cutoff of ≤ 0.001 adjusted for multiple testing using Bonferroni correction (*n*=57,414 SNPs). To focus on ASBs with potential biological relevance, we further restricted ASBs to those located in high-confidence BZR1-binding peaks reproducible in all the biological replicates (*n*=33,267 ASBs). While TFs usually bind small DNA regions of ~10 bp [59], we used 75 bp paired-end sequencing with an average insert size of ~200 bp achieved after sonication. Therefore, SNPs in close proximity with causative polymorphisms will show biased allele frequency due to linkage. To address this, we identified SNPs with significant bias within a 150-bp rolling window of each other and defined the lead SNP with the smallest distance to the peak summit as ASB (*n*=7817). Finally, we excluded ASBs that showed a significant bias (*p*<0.05) in their surrounding region (+/− 1 kb) in the ChIP-input data (i.e., before immunoprecipitation), to avoid potential artifacts (e.g., mapping artifacts or errors in whole genome alignment). To measure bias significance of ASBs and establish an empirical significance threshold, a specific set of control background SNPs were proportionally sampled (excluding ASBs) per chromosome in order to establish a distribution of biases in their surrounding regions (+/− 1000 bp per background SNP). Hence, ASBs whose biases were beyond the upper and lower 5 percentiles (i.e., empirical *p*-value < 0.05) were excluded (*n*=6143 ASBs) from further analysis.

### Gene ontology analysis

Functional enrichment analysis for *Arabidopsis* and Maize BZR1 target genes was performed using the Bingo plugin of Cytoscape (V 3.7.2). *Arabidopsis* identifiers of Maize homologs were used to avoid any bias of the annotation state of the two species.

### Control background SNP sampling

Functional GWAS variants have been shown to be significantly enriched in gene proximal regions [2]. Therefore, control bgSNPs were proportionally sampled (excluding ASBs) per chromosome and genomic location (i.e., 5 - 1 kb upstream, 1 kb upstream - TSS, 5′UTR, exon, intron, 3′UTR, TTS - 1 kb downstream, intergenic) to match the genomic distribution of the ASB dataset. Additionally, we checked that ASBs and bgSNPs showed a similar minor allele frequency (Additional file 2: Fig. S7). In total, 317,094 bgSNPs were sampled, yielding approximately 50 times as many background SNPs per genome location, compared to the number of ASBs within each location.

### Fluorescence imaging

Heterozygous BZR1-YFP plants were grown in the dark at RT for 10 days in vermiculite with and without 10 µM PPZ. Prior to imaging, 1-cm root tip segments were removed from the mock and PPZ-treated seedlings. The root tip of PPZ-treated plants again was treated for 15 min with 10 µM PPZ with or without 1 µM 24epi-BL.

### RNA extraction, RNA sequencing, and differential expression analysis

BR-deficient *brd1* mutant siblings were grown in soil under greenhouse conditions for 26 days as described above. The oldest 2 leaves were removed, and 2 cm of meristem-enriched tissue (Additional file 2: Fig. S1) was placed in 1 µM BL for 4 h at room temperature (RT) in water. Total RNA was isolated using acidic phenol extraction as described previously [60]. Purification of poly-adenylated mRNA using oligo(dT) beads, construction of barcoded libraries, and sequencing using Illumina HiSeq 2500 technology (75 bp paired-end reads) performed by Novogene Co. using the manufacturer’s recommendations. Trimmed and QC (Seqpurge v. 2019-02-11) filtered sequence reads were mapped to B73 AGPv4 using STAR (v. 2.54) [57] in two-pass mode (with parameters: --outFilterScoreMinOverLread 0.3, --outFilterMatchNminOverLread 0.3, --outSAMstrandField intronMotif, --outFilterType BySJout, --outFilterIntronMotifs RemoveNoncanonical, --quantMode TranscriptomeSAM GeneCounts). Unique reads were filtered by mapping quality (q255) and PCR duplicates removed using Samtools (v. 1.3.1). Gene expression was analyzed in R (v. 3.4.1) using the DEseq2 software (v. 1.16.1) [61]. Genes were defined as differentially expressed by a 1.5-fold expression difference with a *p*-value, adjusted for multiple testing, of < 0.05.

For the analysis of gene transcript differences between B73 and Mo17, two parallel data sets were analyzed. First, a previously published RNA-seq data set was used [34] including their differentially expressed genes. Secondly, B73xMo17 F_1_ plants were grown under greenhouse conditions for 21 days as described above. Leaf tissues of three replicates (*n* 12 plants each) were harvested and total RNA extracted using the RNeasy kit (incl. DNAse treatment, Qiagen). The NEB directional Ultra II RNA library kit was used to construct poly-A enriched, barcoded libraries. The default fragment insert size was increased to ~400 bp + adapters, to enhance the yield of reads containing B73/Mo17 variants. Sequencing reads were mapped to the concatenated B73 and Mo17 genome described above using STAR (v.2.7.10a, --outSAMmultNmax 1, --outFilterMultimapNmax 1, --winAnchorMultimapNmax 100, --sjdbOverhang 149, --outFilterIntronMotifs RemoveNoncanonical, --outFilterType BySJout, --twopassMode Basic, --quantMode GeneCounts). The maizegdb pan-gene dataset was used to determine orthologous B73 and Mo17 genes. In contrast to B73v5, almost all Mo17 CAU gene models lacked 5′ and 3′ UTRs. In addition, the increased use of PacBio long-read technology for the B73v5 compared to the Mo17 CAU annotation may explain the B73 bias in our initial allele-specific transcript quantification. To reduce this bias, we standardized 5′ and 3′ UTRs of both B73 and Mo17 genes to 500 bp from the translation start/stop and removed orthologous transcripts with > 50 bp cds length differences from the analysis. Only reads mapping uniquely to B73 or Mo17 and only those overlapping with a single gene model and at least 20 reads in total and at least one read per allele were considered for further analysis. For comparison with ASB genes, genes with 1.5-fold variations in transcript allele frequencies were considered. To further avoid annotation differences in Mo17 and B73 due to the missing UTR annotations in Mo17, ASB genes were annotated to the B73 allele only in this case and then their mRNA abundance in the respective B73/Mo17 pair was considered.

### Genomic feature profiling of ASBs (methylation, motifs, and enhancers)

Inbred methylation levels for CG, CHG, and CHH for B73 and Mo17 were extracted from Regulski et al. [27]. Low-quality reads and eventual adapter contaminants were filtered from inbred and hybrid data by Trimmomatic (version 0.39) [62] and Trim Galore (https://github.com/FelixKrueger/TrimGalore), respectively. Both inbred and hybrid methylation data was mapped to the respective genomes (B73 v5, Mo17 CAU and the diploid hybrid genome) using Bismark (v0.22.3) [63] with bowtie2 (2.4.4) [64] as mapper allowing only unique mapping, methylation counts were extracted with Bismark using the -CX option of the bismark methylation extractor. Resulting CX reports were manually converted into bedgraph format using awk and converted to bigwig format using bedGraphToBigWig [65]. Methylation frequency versus distance (up to +/− 2 kbp) around each ASB were averaged over 10-bp bins, and visualized by regions bound by BZR1 with either high or low affinity levels depending on the inbred line. For B73, high- and low-affinity bound regions were defined by a post frequency of ≥ 0.85 or ≤ 0.15, respectively and oppositely for Mo17 by a %B73 binding frequency (B73/(B73+Mo17)) ≤ 0.15 and ≥ 0.85, respectively.

For local motif enrichment analysis (Fig. 2E), we extracted +/− 100 bp of the high-affinity BZR1 bound allele surrounding ASBs. The MEME CentriMo suite (v. 5.3.3) was used to determine the local distribution along the 201-bp fragments for the canonical BZR1 motifs BRRE (CGTG[T/C]G, C[G/A]CACG) or G-box (CACGTG) and a control motif, with SBP ("GTACGG", "CCGTAC") [51], with a similar GC content.

To identify ASBs overlapping with motif variation (Fig. 2h), we extracted the +/− 5 bp of the high-affinity BZR1 bound allele surrounding ASBs. Using Meme-suit Centrino (v.5.3.3), we scanned those 11-bp fragments for canonical BRRE (CGTG[T/C]G, C[G/A]CACG) or G-box (CACGTG) motifs and determined ASBs where the SNP changed a BRRE or G-box motif into an altered (non BRRE or G-box) motif.

To identify ASBs which overlapped with significant variation in either CG, CHG, or CHH methylation between B73 and Mo17, we first, per ASB, assigned averaged methylation levels of Mo17 and B73 methylation levels (separately for the CG, CHG, or CHH methylation datasets) within a given window of +/− 20 bp around the ASB position. Differentially methylated alleles were defined as described previously [27]. Accordingly, we defined ASBs as overlapping with differentially methylated regions if the B73 or Mo17 methylation level in F_1_ hybrids or inbreds, depending on the analysis, of one allele was ≥ 70% while the level of the corresponding allele was ≤ 10%.

Putative B73 enhancer (Fig. 4a) regions were extracted from Oka et al. [29] and uplifted to B73 AGPv5 using NCBI Remap. To determine potential enrichments, +/− 10 kbps surrounding enhancer regions were intersected with ASBs and bgSNPs.

### GWAS enrichment, kinship matrices, and variance components analysis

We tested association of ASBs with the curated 4041 significant GWAS hits for 41 different phenotypes of the NAM population [2]. Per trait, we performed an enrichment between the ASBs compared to the control bgSNPs (with the same average genomic distribution and minor allele frequency) (Additional file 2: Fig. S7). GWAS hits were counted if they were located within 2 kb of ASBs or bgSNPs, and the subsequent enrichment analysis was based on a hypergeometric test.

We estimated the variance components explained by different ASB SNP subsets and the remaining SNPs using the maize NAM population [38]. To conduct the analysis, we downloaded the phenotypic data (/iplant/home/glaubitz/RareAlleles/genomeAnnos/VCAP/phenotypes/ NAM/familyCorrected), consisting of Best Linear Unbiased Predictors (BLUPs) for different traits [2], and the imputed genotypic data (/iplant/home/glaubitz/RareAlleles/genomeAnnos/VCAP/genotypes/NAM/namrils_projected_hmp31_MAF02mnCnt2500.hmp.txt.gz) [66] from CyVerse database as described in Panzea (www.panzea.org). In the analysis, we mapped ASB SNPs and randomly sampled bgSNPs that shared the similar genomic patterns to upstream 5 kb - TSS, within the CDS, TTS - 5 kb downstream of genes as well as intergenic regions. Since the Mo17 CAU annotation often lacks 5′ and 3′ UTRs, both the ASB and bgSNP positional annotation for VCAP was based on their B73 coordinates. The bgSNPs were resampled based on the ASB distribution for a total of 254,017 VCAP-bgSNPs. For each SNP subset, we calculated an additive kinship matrix using the variance component annotation pipeline (VCAP) implemented in TASSEL5 [67]. We then fed these kinship matrices along with the NAM phenotypic data to estimate the variance components explained by the ASB subsets, using a Residual Maximum Likelihood (REML) method implemented in LDAK [68].

Co-localization of candidate genes within joint linkage QTLs for resistance to NLB and SLB was represented graphically using R version 3.2.3 (R Core Team, 2015). The start and end sites of NAM QTL were identified in AGPv2 for NLB [69] and SLB [70]. Corresponding AGPv2 locations of candidate genes were identified via maizegdb.org [71].

### Northern leaf blight assay

A 2 × 2 factorial experimental design included three replications, B73 and Mo17 genotypes, and two treatments (with or without PPZ) within each replication. B73 and Mo17 were planted (n>7) in 10 cm pots (1 plant per pot) in the greenhouse. At the V6-stage, the plants were treated with either 400 µM PPZ or mock solution through soil fertilizer drenching every 3 to 4 days. Four days after the initial PPZ treatment, the plants were inoculated with a 500-µl liquid spore suspension of *Setosphaeria turcica* isolate NY001 (race1), containing 4000 spores/ml, into the whorl during the late afternoon. An overhead mister provided water for 10 s every 10 min for approximately 15 h. Disease phenotype was measured as the incubation period (IP), the number of days following inoculation when a necrotic lesion was first observed. Forty-eight days following inoculation, the disease measurements were concluded; a survival analysis was performed in JMP® Pro Version 13.1.0 (SAS Institute, Inc, Cary, NC, 1989–2019) to test for differences within each genotype for the effect of PPZ on IP using a log-rank test statistic and visualized in a Kaplan-Meier plot.

## Supplementary Information

**Additional file 1: Table S1.** List of 2743 BR responsive genes in Maize determined by RNA-seq.

**Additional file 2: Supplementary figures. Fig. S1.** Additional QC data for BZR1-YFP and HASCh-seq. **Fig. S2.** Multiple sequence alignment of BZR1 DNA-binding domain. **Fig. S3.** IP-Input allelic bias control for HASCh-seq. **Fig. S4.** ASBs of ZmBZR1 overrepresentation at enhancer sites. **Fig. S5.** Differential NBL resistance of B73 and Mo17 in response to BR inhibitor PPZ treatment. **Fig. S6.** ASBs overlapping with complex differences in ZmBZR1 binding pattern. **Fig. S7.** ASBs and bgSNPs share similar minor allele frequency.

**Additional file 3: Table S2.** List of 17463 putative ZmBZR1 binding sites of the B73 inbred line.

**Additional file 4: Table S3.** List of 6371 ZmBZR1 target genes. Target genes were defined if the gene CDS +5 kb upstream or -1 kb downstream overlapped with a ZmBZR1 peak.

**Additional file 5: Table S4.** List of 2058 conserved BRZ1 target genes in Maize with orthologs in *Arabidopsis.*

**Additional file 6: Table S5.** List of GO-slim plant terms found enriched in Maize only BZR1 target genes, *Arabidopsis* only BZR1 target genes or both. Significantly over-enriched terms in the respective category are marked by a green background, non-enriched ones in either *Arabidopsis* only or maize only are marked in red, under-enriched terms are marked by red font color of the p-values. For comparison, p-values and enrichment of shared BZR1 target genes are displayed for the same categories.

**Additional file 7: Table S6.** List of 52765 ZmBZR1 binding sites reproducible in all 6 replicas of B73xMo17 F1 hybrids and genes annotated to these regions. Genes with significant BZR1 binding sites from 5 kb upstream to 1 kb downstream of the TSS and TTS, respectively, were considered target genes.

**Additional file 8: Table S7.** List of ZmBZR1 target genes in B73xMo17 hybrids. Genes with significant BZR1 binding sites from 5 kb upstream to 1 kb downstream of the TSS and TTS, respectively, were considered target genes. Gene names are given in B73 and Mo17 coordinates where a homologous gene exists, otherwise only one gene is listed.

**Additional file 9: Table S8.** List of 33267 SNPs with significant allele-specific bias in linkage groups.

**Additional file 10: Table S9.** Genomic location of the 6143 lead SNPs of allele-specific BZR1 binding site, genomic locations of ASBs and association with genes at the high-affinity allele.

**Additional file 11: Table S10.** List of putative ASB ZmBZR1 target genes. Genes with significant BZR1 binding sites from 5 kb upstream to 1 kb downstream of the TSS and TTS, respectively, were considered target genes. Gene names are given in B73 and Mo17 coordinates where a homologous gene exists, otherwise only one gene is listed.

**Additional file 12: Table S11.** ASBs overlapping with variations in motifs and/or DNA methylation.

**Additional file 13: Table S12.** Raw RNA-seq counts in the B73xMo17 hybrids for genes with homologs in both genome annotations. Fold-changevalues were calculated for each replicate and an average FC was calculated to be employed for further analysis. For comparison with ASB genes, only genes with differences in CDS length <=50bp between B73 and Mo17 were employed.

**Additional file 14: Table S13.** ASBs co-locolized within 2 kb of the 4015GWAS hits from Wallace et al. 2014

**Additional file 15: Table S14.** Primers employed in this study.

**Additional file 16: Table S15.** F1 samples sequencing statistics.

**Additional file 17: Table S16.** MACS3 HASCh-seq peaks of reads that map uniquely to one or both parents. BZR1-YFP replicates vs. non-transgenic negative control were used for the peak calling.

**Additional file 18.** Review history.

## Data Availability

All HASCh-seq, ChIP-seq, B73xMo17 eMethyl-seq, and RNA-seq data discussed in this publication have been deposited at NCBI SRA under accession number PRJNA906943 [72]. HASCh-seq custom scripts have been deposited in the Github repository https://github.com/mbanf/HaschSeq under the GNU General Public License v3.0 [73]. Inbred B73 and Mo17 DNA methylation data previously published [74] was re-analyzed for B73 AGPv5 and Mo17 CAU genomes. Other scripts and software used in this study are included in the “Methods” section.
