# Supplementary material for: Hybrid allele-specific ChIP-seq analysis identifies variation in brassinosteroid-responsive transcription factor binding linked to traits in maize
Source: Genome Biol. 2023 May 8;24:108. doi: 10.1186/s13059-023-02909-w (PMC10165856; doi:10.1186/s13059-023-02909-w)
Supplement: Supplementary file 2 — Additional file 2: Supplementary figures. Fig. S1. Additional QC data for BZR1-YFP and HASCh-seq. Fig. S2. Multiple sequence alignment of BZR1 DNA-binding domain. Fig. S3. IP-Input allelic bias control for HASCh-seq. Fig. S4. ASBs of ZmBZR1 overrepresentation at enhancer sites. Fig. S5. Differential NBL resistance of B73 and Mo17 in response to BR inhibitor PPZ treatment. Fig. S6. ASBs overlapping with complex differences in ZmBZR1 binding pattern. Fig. S7. ASBs and bgSNPs share similar minor allele frequency. [file 13059_2023_2909_MOESM2_ESM.docx]

**Additional file 1:** Supplementary figures (Fig. S1-S7) of Hybrid allele-specific ChIP-seq analysis identifies variation in brassinosteroid-responsive transcription factor binding linked to traits in maize.





**Fig. S1: Additional QC data for BZR1-YFP and HASCh-seq.** **a)** Influence of BL and propiconazole (PPZ) on ZmBZR1 nuclear localization. **b)** ChiP qPCR to verify ChIP-seq results at *URL2,* *BR6ox2* and *BRI1-1* loci. **c)** Comparison of zmBZR1 target genes with AtBZR1 target genes obtained by ChIP-chip on 4-week old light grown rosettes^[1](https://www.zotero.org/google-docs/?r7M9hY)^  and ChIP-seq on 5-day-old dark grown seedlings^[2](https://www.zotero.org/google-docs/?yYqePw)^ **d)** Pearson correlation coefficients between replicates for inputs and BZR1 ChIP in B73xMo17 (rep1-3) and Mo17xB73 (rep4-6) **e)** Principal component analysis of the six replicates for ChIP and input **f)** Example of tissue dissection performed in this study. The meristem-enriched tissue employed for RNAseq and ChIPseq in this study is indicated by a red square. g) Western blot of YFP-tagged BZR1 detected by GFP1 antibody: No background signal was observed for control plants without the ZmBZR1-YFP construct. FT=Flowthrough; IP= Immunoprecipitation step.

**AtBZR1** MTSDGATSTSAAAAAAAAAAARRKPSWRERENNRRRERRRRAVAAKIYTGLRAQGDYNLP **60**

**B73-BZR1** MTS---------G-AAAAGGLGRTPTWKERENNKRRERRRRAIAAKIFTGLRALGNYKLP **50**

**Mo17-BZR1** MTS---------GAAAAAGGLGRTPTWKERENNKRRERRRRAIAAKIFTGLRALGNYKLP **51**

*** ..****.. *.*:*:*****:********:****:***** *:*:**

**AtBZR1** KHCDNNEVLKALCVEAGWVVEEDGTTYRKGCKPLPGEIAGTSSRVTPYSSQNQSPLSSAF **120**

**B73-BZR1** KHCDNNEVLKALCREAGWVVEDDGTTYRKGCRPPPGMLSPCSS------SQLLSAPSSSF **104**

**Mo17-BZR1** KHCDNNEVLKALCREAGWVVEDDGTTYRKGCRPPPGMLSPCSS------SQLLSAPSSSF **105**

************* *******:*********:* ** :: ** ** * **:*

**Fig. S2: Multiple sequence alignment of BZR1 DNA-binding domain.** Ammino acid alignment of BZR1 Arabidopsis Col.0 and Zea mays B73 and Mo17.


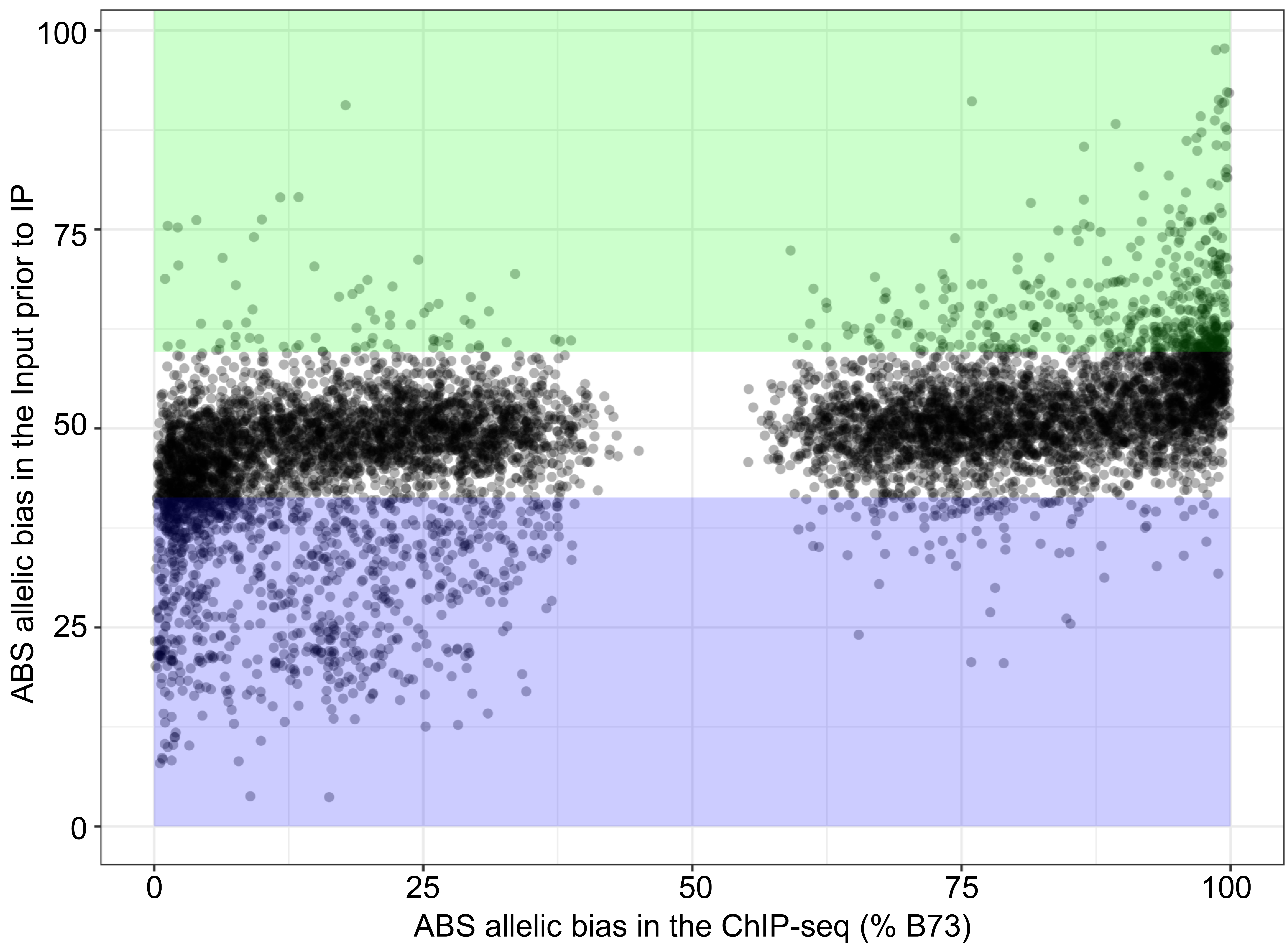


ASB allelic bias in the HASCh-seq data (% B73)

**Fig. S3: IP-Input allelic bias control for HASCh-seq.** Filtering of ASBs which showed a significant bias in their surrounding area prior to immuno-precipitation in the input data. Allelic bias (% B73) of ASBs in the Input (y-axis) and HASCh-seq (x-axis). Green and Blue zones depict significant (< 0.05) deviation from the expected 0.5 B73/Mo17 allelic ratio.

**Fig. S4: ASBs of ZmBZR1 overrepresention at enhancer sites.** ASB and bgSNP density around intergenic enhancer sites +/- 10 kb.

**Fig. S5: Differential NBL resistance of B73 and Mo17 in response to BR inhibitor PPZ treatment.** Kaplan-Meier survival plots of B73 (left) and Mo17 (right) parents treated with or without *Setosphaeria turcicum* and with and without co-treatment of the BR biosynthesis inhibitor PPZ. As expected, under Mock conditions necrotic lesions appeared later after infection for Mo17 compared to B73 plants. PPZ treatment significantly increased the incubation time needed for necrotic lesion formation for B73 plants, and resulted in no observed lesion formation, within the assay time, for Mo17.

**Fig. S6: ASBs overlapping with complex differences in ZmBZR1 binding pattern.** Allele-specific, cumulative HASCh-seq B73 (green) or Mo17 (blue) signal around ASBs that co-localized with GWAS hits for tassel length, cob length, stalk strength, and total amino acids near *ZmSTP4* (Zm00001eb324180).





**Fig. S7: ASBs and bgSNPs share similar minor allele frequency.** Minor allele frequencies of ASBs (red) and bgSNPs (blue).
