# Supplementary material for: Hybrid allele-specific ChIP-seq analysis identifies variation in brassinosteroid-responsive transcription factor binding linked to traits in maize
Source: Genome Biol. 2023 May 8;24:108. doi: 10.1186/s13059-023-02909-w (PMC10165856; doi:10.1186/s13059-023-02909-w)
Supplement: Supplementary file 18 — Additional file 18. Review history. [file 13059_2023_2909_MOESM18_ESM.docx]

Review History

**First round of review**

**Reviewer 1**

**Were you able to assess all statistics in the manuscript, including the appropriateness of statistical tests used?**

No, I do not feel adequately qualified to assess the statistics.

**Comments to author:**

This manuscript explores the potential impact of allelic differences between two widely used maize inbred lines (B73 and Mo17) in the binding and regulation of targets of the brassinosteroid responsive transcription factor BZR1. The authors present the study as a novel application of ChIP-seq (hybrid allele-specific ChIP-Seq, or HASCh-seq) to identify allele-specific binding (ASB) sites. The method however is not really novel and has been used already in metazoans. The ASB results were then compared with allele specific expression of BZR1 targets to identify the genes in which differential BZR1 binding might be responsible for the different expression of the alleles. RNA-Seq results were further validated by qRT-PCR; however no independent demonstration of the ASB (e.g., by EMSA) was provided. Finally, the authors correlate the ASB sites with GWAS data, suggesting that ASBs are responsible for phenotypic diversity - interesting possibility, but which requires more robust validation of the BZR1 binding sites.

Overall, the study is of interest but a number of technical concerns, over-interpretations of the data and unreasonably overstatement of the novelty made me less enthusiastic about the study.

Comments

* The ZmBZR1-YFP fusion expressed from the putative ZmBZR1 promoter was not shown previously to complement bzr1 mutations, or fully recapitulate BZR1 expression, hence results of the ChIP-Seq must be taken with care, both from the perspective of the expression domain of the promoter and the activity of the fusion protein

* What was the negative control in the ChIP-Seq experiments? I may have missed it, but were GFP antibodies used on wild type plants, or even better on plants expressing YFP from the putative BZR1 promoter? Input is not an appropriate negative control for ChIP-Seq.

* In this regard, it would have been valuable to obtain some statistics of the ChIP-Seq results - total number of reads for experiment and controls, % alignment, % duplicated eliminated, % multi-mapping and an explanation of how the authors dealt with them, etc.

* Lane 339: Unclear how reproducible peaks were called - they needed to be within the 400 bp window? If so, that is not a very strong requirement.

* Without knowing how deep the input data was sequenced, it is impossible to assess whether all the regions of systematic bias (Lanes 349-351) were identified. This is very important for the determination of allele binding preferences.

* The source of the B73 and Mo17 maize materials must be reported - as the authors are surely aware, there are a number of changes between different stocks that can significantly impact results.

* In Fig. 1b, are the ChIP reads normalized somehow? As shown, it is misleading as the background signal is not evident

* The entire first part of the manuscript is essentially a repetition of experiments done in Arabidopsis, but now in maize. Not surprisingly, many similar targets were found - what about the differences? I wonder if that is where the new interesting biology might be.

* Unclear why B73 and Mo17 were selected - any evidence that they respond differently to BR signaling?

* How were the repeat ChIp-Seq in the Mo17 x B73 or B73 x Mo17 carried out? Just one plant or three different plants in each genotype? Given that the ZmBZR1-YFP/B73 line is likely to still harbor many regions derived from the HiII hybrid in which the transgenics were originally generated.

* There is a significant flaw in the analysis of the allelic ChIP-Seq, and that is that the authors are assuming equal representation of both alleles for each gene in the input library. This can only be tested by sequencing the input library at enough depth to be able to do robust statistical analyses. In our experience, there is significant difference of the representation of alleles in genomic libraries used for ChIP-Seq (or DAP-Seq).

* The proposed effects of methylation on BZR1 DNA-binding preference are correlative and no effort was made to validate them. This is exemplified by the positive correlation of BZR1 DNA binding and CHH methylation, which the authors attributed to an indirect effect of CHH methylation and high expression, which characterizes (at least some) BZR1 targets.

* The proposed correlation between allele-specific binding and differential allele expression must be better described. For example, in how many instances was increased allelic binding associated with increased expression, and in how many instances was this the other way around. Demonstrating directly how DNA-binding (CpG, CNG, CHH) affects BZR1 binding is essential to make sense of the observations.

**Reviewer 2**

**Were you able to assess all statistics in the manuscript, including the appropriateness of statistical tests used?**

No, I do not feel adequately qualified to assess the statistics.

**Comments to author:**

This study describes a hybrid allele-specific chromatin binding sequencing (HASCh-seq) method for quantifying the differential binding of ZmBZR1 to the B73 and Mo17 genomes. The authors identified 3395 independent, allele-specific BZR1 binding sites, over 550 of them harbor variations in BZR1-bindimng motifs and over 340 loci show haplotype-specific DNA methylation. Compared to previous GWAs data, the authors established a link between these variations with phenotypic diversity of maize. The approach used in this study is novel and exciting, which nicely complements other approaches routinely used in quantitative genetics and molecular studies.

I have a few suggestions for the authors to consider for improving the quality of this work and solidify their conclusions:

1. A major concern is that the authors generated a pseudo Mo17 genome based on the B73 AGPv4 genome assembly. High-quality genome of Mo17 has become available in 2018 (Sun et al., Nature genetics, 50:1289-1295), which also reports extensive genetic variations between Mo17 and B73. I worry that the pseudo Mo17 genome used in this study would cause substantial bias towards SNP calling. Quality control is essential.

2. The genome-wide methylation data of B73 and Mo17 was derived from a previous study with inbred lines (Regulski, et al., 2013), which is different from the B73 x Mo17 F1 hybrids used for HASCh-seq in this study. This could represent a potential problem as the methylation pattern could change in the F1 versus the inbred lines, not mentioning the tissues /experimental conditions used in these studies might differ.

3. How the allele-specific transcriptome data of B73 x Mo17 F1 hybrids was generated? Any experimental validation has been performed?

4. For linking the sequence variation to phenotype, the specific NAM population should be explained. They found enrichment of ASBs for a number of traits. It will be nice if the authors can dig into the data a bit more, and provide a case study to establish a direct link between the target gene and trait variation between B73 and Mo17.

5. The advantage and potential pitfalls of HASCh-seq should be discussed and elaborated in the discussion, as maize is known to have strong heterosis. The differential binding of ZmBZR1 to B73 and Mo17 genome in the F1 background may not faithfully reflect the situation in the respective inbred lines. For example, the used allele-specific expression data from a previous study (Baldauf et al., 2016). It is well known that in the F1 hybrids, some genes show additive expression, while others may show non-additive, parental-specific expression due to various mechanisms (genetic interaction, epigenetic regulation etc.). The pitfalls of such approaches have to be properly acknowledged.

6. To be informative, in the supplementary tables1-4, gene annotation of Arabidopsis and maize should be given, and genes with functional studies should be referenced.

**Authors Response**

**Point-by-point responses to the reviewers’ comments:**

Reviewer #1:

This manuscript explores the potential impact of allelic differences between two widely used maize inbred lines (B73 and Mo17) in the binding and regulation of targets of the brassinosteroid responsive transcription factor BZR1. The authors present the study as a novel application of ChIP-seq (hybrid allele-specific ChIP-Seq, or HASCh-seq) to identify allele-specific binding (ASB) sites. The method however is not really novel and has been used already in metazoans. The ASB results were then compared with allele specific expression of BZR1 targets to identify the genes in which differential BZR1 binding might be responsible for the different expression of the alleles. RNA-Seq results were further validated by qRT-PCR; however no independent demonstration of the ASB (e.g., by EMSA) was provided. Finally, the authors correlate the ASB sites with GWAS data, suggesting that ASBs are responsible for phenotypic diversity - interesting possibility, but which requires more robust validation of the BZR1 binding sites.

Overall, the study is of interest but a number of technical concerns, over-interpretations of the data and unreasonably overstatement of the novelty made me less enthusiastic about the study.

*Response: We have addressed the technical concerns to the best of our ability, tuned down our interpretations, and used more precise language to describe our work and its novelty. These include a change of our title from HASCh-seq “links variation to traits” to HASCh-seq “identifies variations” which are linked to traits. Lastly, we added ENCODE efforts which developed genome-wide maps of variants affecting TF binding in mammalian systems in the introduction.*

Comments

* The ZmBZR1-YFP fusion expressed from the putative ZmBZR1 promoter was not shown previously to complement bzr1 mutations, or fully recapitulate BZR1 expression, hence results of the ChIP-Seq must be taken with care, both from the perspective of the expression domain of the promoter and the activity of the fusion protein.

*Response: There is no bzr1 mutant in maize. In Arabidopsis, the single bzr1 loss-of-function mutant shows no phenotype. Knocking out five BZR1 homologs resulted in dwarf and sterile phenotypes. However, as we discussed in the Introduction section, previous studies have provided many lines of evidence supporting the conserved function of ZmBZR1, activity of its YFP fusion, and ZmBZR1-YFP’s expression in BR-responsive cells. For example, Kir et al (2021) showed that in this ZmBZR1-YFP transgenic line, the ZmBZR1-YFP protein showed nuclear accumulation in BR-responsive tissues, the nuclear signal was decreased in zmbri1-RNAi lines, increased by BR treatment in wild-type background but less responsive to BR in the zmbri1-RNAi background. Another study showed that overexpression of a maize BZR1 homolog in Arabidopsis caused large-organ phenotypes similar to those caused by BR treatment or overexpression of BR biosynthetic gene or BR receptor BRI1 (Zhang et al., 2020). These studies together provide sufficient evidence for ZmBZR1’s conserved role as the BR signaling transcription factor in maize and the functionality of ZmBZR1-YFP. Our study focuses on the genetic variation based on relative ZmBZR1 occupation of different alleles in the F1 line.*

* What was the negative control in the ChIP-Seq experiments? I may have missed it, but were GFP antibodies used on wild type plants, or even better on plants expressing YFP from the putative BZR1 promoter? Input is not an appropriate negative control for ChIP-Seq.

*Response: For the inbred BZR1 ChIP, we used a negative control with the anti-GFP antibody using non-GFP harboring siblings of ZmBZR1-YFP. We changed the wording in the methods accordingly. We further included the track of the negative control in Fig. 1b. We now further include a negative control with the anti-GFP antibody employed on non-GFP harboring siblings of ZmBZR1-YFP/B73xMo17 treated the same way as for IP (including BR treatment). We do not have a BZR1::YFP transgenic maize (expressing YFP from BZR1 promoter). The negative control was used to redo the peak calling, which is also the basis for ASB calling.*

* In this regard, it would have been valuable to obtain some statistics of the ChIP-Seq results - total number of reads for experiment and controls, % alignment, % duplicated eliminated, % multi-mapping and an explanation of how the authors dealt with them, etc.

*Response: We added a supplementary table 15 that shows some of these statistic information. We clarified in the methods that only uniquely mapping reads were used.*

* Lane 339: Unclear how reproducible peaks were called - they needed to be within the 400 bp window? If so, that is not a very strong requirement.

*Response: Thank you for pointing out that this was mis-presented in the manuscript. The reproducible peaks were called when a peak’s summit is within the +/-200-bp window of another peak summit. Therefore, the window is 199 bp. We explain this in more detail now.*

* Without knowing how deep the input data was sequenced, it is impossible to assess whether all the regions of systematic bias (Lanes 349-351) were identified. This is very important for the determination of allele binding preferences.

*Response: Thanks for the comment. Indeed our original sequencing of the input was not deep enough (10x). We have performed additional sequencing for the input to increase the coverage to about 40x. This deeper sequence allowed us to exclude ASBs that showed bias in the input within their surrounding area, we describe the process in the methods and added Supplemental Fig. 2.*

* The source of the B73 and Mo17 maize materials must be reported - as the authors are surely aware, there are a number of changes between different stocks that can significantly impact results.

*Response: We thank the reviewer for the reminder. We have included the information in the material.*

* In Fig. 1b, are the ChIP reads normalized somehow? As shown, it is misleading as the background signal is not evident

*Response: As mentioned above, we now updated this figure and included the track of the negative control.*

* The entire first part of the manuscript is essentially a repetition of experiments done in Arabidopsis, but now in maize. Not surprisingly, many similar targets were found - what about the differences? I wonder if that is where the new interesting biology might be.

*Response: We agree that this is a very interesting point. We now included a GO analysis where we compared GO annotations of the Arabidopsis-only and maize-only BZR1 targets (Supl.Tab S5). Most of the GO enriched in the species-unique datasets are also enriched in the shared targets. However, a small number of functions were found to be unique in maize, including lipid metabolism. The analysis suggests that different genes involved in the same function are targets in the two species, suggesting conservation of function with divergence of mechanisms.*

* Unclear why B73 and Mo17 were selected - any evidence that they respond differently to BR signaling?

*Response: There was no previous evidence that B73 and Mo17 respond differently to BR. However, they show differences in many phenotypes that are affected in BR mutant maize, such as plant height, tassel branching, flowering time, leaf width, and leaf angle. BZR1 regulates thousands of genes and many developmental processes and the variations in BZR1-binding cis-elements may not cause obvious differences in BR responses but can be QTLs that contribute to specific phenotype variations that can only be detected in focused phenotype analysis. We now included two sentences in the manuscript to explain our choice of hybrid: “We chose B73 and Mo17 as they are among the most diverse maize inbred lines and their hybrid is one of the most studied [24–26]. They also show differences in many phenotypes that are affected in BR mutant maize, such as plant height, tassel branching, flowering time, leaf width, and leaf angle [24,26].”*

* How were the repeat ChIp-Seq in the Mo17 x B73 or B73 x Mo17 carried out? Just one plant or three different plants in each genotype? Given that the ZmBZR1-YFP/B73 line is likely to still harbor many regions derived from the HiII hybrid in which the transgenics were originally generated.

*Response: Thank you for pointing this out. We have clarified this now in the method section by adding: “To back cross ZmBZR1-YFP from its original HiII (B73xA188) background, transgenic lines were used as pollen and B73 as ear donor to eliminate cross contamination. Six backcross lines were independently backcrossed six times, using heterozygous to avoid gene silencing. Lastly, B73-BC5 lines were used as pollen and ear donors for Mo17xB73BZR1-YFP and B73BZR1-YFPxMo17 crosses, respectively. For allele-specific analysis tissues from 12 plants were pooled per replicate. The residual HiII regions cannot be completely removed but were minimized by the many backcrosses and sample pooling and further addressed by our deep sequencing of the input.”*

*There is a significant flaw in the analysis of the allelic ChIP-Seq, and that is that the authors are assuming equal representation of both alleles for each gene in the input library. This can only be tested by sequencing the input library at enough depth to be able to do robust statistical analyses. In our experience, there is significant difference of the representation of alleles in genomic libraries used for ChIP-Seq (or DAP-Seq).

*Response: We thank the reviewer for these critical comments. We agree that unequal presence of alleles in the input is a source of error. We have performed more sequencing of the input to reach about 40x genome coverage. Using the input sequencing data we exclude the ASBs that showed a significant input deviation from the expected 1:1 distribution in their surrounding area. The details of experiment and data analysis have been added in Materials and Methods and in Supplemental Fig. S2. All figures now reflect the new ASB datasets.*

*The proposed effects of methylation on BZR1 DNA-binding preference are correlative and no effort was made to validate them. This is exemplified by the positive correlation of BZR1 DNA binding and CHH methylation, which the authors attributed to an indirect effect of CHH methylation and high expression, which characterizes (at least some) BZR1 targets.

*Response: We agree with the reviewer that what we observed is restricted to correlation. However, the opposite correlations observed for CG/CHG and CHH are consistent with their known different effects on gene expression. We now clarify that about 99% of the ASB associated methylation events are CG and CHG, while CHH methylation constitutes only about 1% (18 ASBs). Further, we performed Methyl-seq data in F1 treated with BR (to match ChIP conditions) and the results show a stricker correlation between CG/CHG methylation and decreased BZR1 binding (99.2%), and no obvious difference to data from plants untreated with BR, suggesting that BR does not obviously affect CG/CHG methylation. We did not make efforts to validate the effects of methylation on BZR1 DNA-binding partly because DAP-seq analysis in Arabidopsis has shown that the DNA binding of BES1/BZR2, and that of its partners ARFs, is inhibited by DNA methylation. Nevertheless, in response to this reviewer’s comments, we have performed DAP-seq twice with ZmBZR1 and native genomic DNA and PCR amplified methylation-free DNA, but the results were of poor quality and not conclusive. Given the high conservation between ZmBZR1 and AtBZR1 (78% identity in the DNA-binding domain, and binding to the same BRRE/G-box motif), we think it is a safe assumption that the DNA binding of ZmBZR1 is like AtBZR2 also sensitive to DNA methylation.*

* The proposed correlation between allele-specific binding and differential allele expression must be better described. For example, in how many instances was increased allelic binding associated with increased expression, and in how many instances was this the other way around.

*Response: We now generated RNA-seq data in B73xMo17 hybrids and added the numbers of instances of ASB genes up- and downregulated in (Fig 3c). As expected for BZR1, which is known to act as an activating or repressing TF for different target genes (also Fig 1d), BZR1 binding is correlated with increased expression of some genes and decreased expression of others. The correlation between allele-specific binding and differential expression was also supported by the high overlap between the ASB genes and genes differentially expressed between B73 and Mo17 inbreds and in the newly added hybrid data (Fig 3a and 3b), as well as the qRT-PCR analysis of selected ASB genes (Fig 3f and 3i).*

* Demonstrating directly how DNA-binding (CpG, CNG, CHH) affects BZR1 binding is essential to make sense of the observations

*Response: We presume you mean demonstrating how DNA methylation affects BZR1 binding? We performed DAP-seq of ZmBZR1 using native and PCR amplified genomic DNA, but the results were not conclusive. On the other hand, DAP-seq analysis in Arabidopsis showed that the DNA binding of BES1/BZR2, and that of its partners ARFs, is inhibited by DNA methylation. Therefore, it makes sense that ZmBZR1 would show methylation-sensitive binding in vivo.*

Reviewer #2:

This study describes a hybrid allele-specific chromatin binding sequencing (HASCh-seq) method for quantifying the differential binding of ZmBZR1 to the B73 and Mo17 genomes. The authors identified 3395 independent, allele-specific BZR1 binding sites, over 550 of them harbor variations in BZR1-bindimng motifs and over 340 loci show haplotype-specific DNA methylation. Compared to previous GWAs data, the authors established a link between these variations with phenotypic diversity of maize. The approach used in this study is novel and exciting, which nicely complements other approaches routinely used in quantitative genetics and molecular studies.

I have a few suggestions for the authors to consider for improving the quality of this work and solidify their conclusions:

1. A major concern is that the authors generated a pseudo Mo17 genome based on the B73 AGPv4 genome assembly. High-quality genome of Mo17 has become available in 2018 (Sun et al., Nature genetics, 50:1289-1295), which also reports extensive genetic variations between Mo17 and B73. I worry that the pseudo Mo17 genome used in this study would cause substantial bias towards SNP calling. Quality control is essential.

*Response: We thank the reviewer for this suggestion. We completely revised our mapping strategy and are now using the two fully assembled genomes B73 AGPv5 and Mo17-CAU as suggested by the Reviewer. We also added a step that filtered ASBs which showed a bias in the INPUT data. This new analysis resulted in a more even overall allelic distribution (50.08% B73 alleles). Furthermore, we identified more than twice as many ASBs, yet their overlap with BZR1 motifs increase from 16% to 23%. We added Figure 2c to demonstrate the lack of mapping bias and allelic distribution of the data.*

2. The genome-wide methylation data of B73 and Mo17 was derived from a previous study with inbred lines (Regulski, et al., 2013), which is different from the B73 x Mo17 F1 hybrids used for HASCh-seq in this study. This could represent a potential problem as the methylation pattern could change in the F1 versus the inbred lines, not mentioning the tissues /experimental conditions used in these studies might differ.

*Response: We agree with the reviewer that there can be differences in DNA methylation between parental lines and F1 hybrid, although previous studies showed that most DNA methylation patterns are preserved in Arabidopsis. We therefore performed EM-methyl-seq for B73xMo17 hybrids treated similarly to the ChIP samples. We include a new methods paragraph “EM-methyl-seq” which describes the experimental details. We include Figure 2i to show the similarities between inbred and F1 methylation data at ASB sites. The F1 methylation data showed a stronger correlation (99.2%) between methylation and reduced ZmBZR1 binding. We updated the main figures with the F1 methylation data.*

3. How the allele-specific transcriptome data of B73 x Mo17 F1 hybrids was generated? Any experimental validation has been performed?

*Response: The allele-specific transcriptome data originally used was from the inbred lines (Baldauf et al. 2016). We have clarified this info in the revised manuscript. We have also performed RNA-seq in B73 x Mo17 F1 hybrids and added this data as well as described in the response to reviewer 1.*

4. For linking the sequence variation to phenotype, the specific NAM population should be explained. They found enrichment of ASBs for a number of traits. It will be nice if the authors can dig into the data a bit more, and provide a case study to establish a direct link between the target gene and trait variation between B73 and Mo17.

*Response: This was a very valid point raised and we now include details of several examples of disease genes where ASBs locate close to known GWAS hits (Figure 5, Supplemental Fig. S5). We added a short paragraph describing the maize NAM population and added an appropriate citation.*

5. The advantage and potential pitfalls of HASCh-seq should be discussed and elaborated in the discussion, as maize is known to have strong heterosis. The differential binding of ZmBZR1 to B73 and Mo17 genome in the F1 background may not faithfully reflect the situation in the respective inbred lines. For example, the used allele-specific expression data from a previous study (Baldauf et al., 2016). It is well known that in the F1 hybrids, some genes show additive expression, while others may show non-additive, parental-specific expression due to various mechanisms (genetic interaction, epigenetic regf

*Response: We do agree that the situation in the hybrids might be very different compared to the inbreds. We therefore updated the discussion accordingly: “ASBs identified in the F1 reflect the variations of DNA sequence and methylation between the two haplotypes, however, the trans factor environment of the F1 may not be identical to either parent. The effect of a motif variation on BZR1 binding is likely similar in the F1 and parental lines, yet the impact on gene expression and phenotype would be confounded by other factors that are different in the F1 and the two parents. This is especially expected in plants which show strong heterosis, such as maize. Similarly, phenotype data used for GWAS predominantly comes from inbred lines. While the DNA sequence and most DNA methylation status are maintained in the F1, some epigenetic differences such as chromatin status between parental lines might be lost in the F1 and thus missed by HASCh-seq but detected in the parental line. ”*

6. To be informative, in the supplementary Tables S1-4, gene annotation of Arabidopsis and maize should be given, and genes with functional studies should be referenced.

*Response: We now added more information to these Tables, including genome V5 annotation, gene names and protein functions. We cited references for genes that we mentioned in the text.*

*We hope that you are satisfied with our extensive revision.*

**Second round of review**

**Reviewer 2**

In this revised manuscript, the authors have conducted additional experiments and tried their best to fully address the reviewers' constructive comments, and as a result, the quality is substantially improved and the conclusions are much more solidified. This reviewer appreciates the authors' substantial efforts made to this work. Although some potential pitfalls are still hard to overcome at the current stage, I am satisfied with this revision and it does provide new insights into BR biology in maize.

A few minor suggestions:

1. The sentence in Abstract (Background) "Genetic variation in the BR-responsive cis-element is likely causal to trait variation" could be changed to "Genetic variation in the BR-responsive cis-element likely contributes to trait variation";

2. For RNA-seq, why shoot tissues were used? Explain

3. The statement of B73 has the best genome annotation might not be true anymore with the release of a number dozens of new maize genomes recently (Like Hufford et al., Science, 2021). Please double check.

4. In a number of places, "F1" should be corrected to "F1 (lower case)".

7. Do B73 and Mo17 show trait variation to NLB/SLB resistance/response? Or maybe they can also look at other founder lines of the NAM population to link ASBs to gene expression and trait variation? Do any of the ASBs co-localize or close to any QTLs for NLB/SLB resistance?

**Reviewer 3**

This study reported the allele specific binding profile of ZmBZR1 in a hybrid background. BZR1 was supposed to be key player in maize BR pathway. The author found that the binding affinity of BZR1 is DNA-methylation related and quite some binding sites locate in enhancer regions. More interestingly, they found a significant overlap between allele-specific binding of ZmBZR1 and trait related SNP in the GWAS analysis, indicating the importance of BZR1 binding preference in controlling trait performance. The work is novel in plants and brings the community a nice example to link TF binding and trait variation. However, there are some concerns need to be addressed.

Major concern:

1. The author documented quite some evidence to show that ZmBZR1 is a key regulator to control maize development, but they are indirect evidence. A knock-out mutant will be very important to enhance the biologically meaning of this work. It makes sense to say that the traits are affected by allele specific binding of BZR1 only when you see the trait is altered in the mutant. And, would be very nice to see that the mutant could be rescued by BZR1-GFP. Meanwhile, if a mutant is available, differentially expressed genes between wild type and the mutant would be more relevant to the binding of BZR1 then the BR induced genes.

2. Since the author performed ChIP in F1 background, in which two endogenous BZR1 copies are binding to their targets, better to verify that the binding affinity of BZR1-GFP is comparable to the endogenous ones (their genotype should be investigated). In this regard, complementation of bzt1 mutation by BZR1-GFP would help.

3. line 243-250, the author showed that a certain proportion of genes with ASB showed allele-specific gene expression both between B73 and Mo17 and in B73xMo17 hybrids. However, this should be more due to the sequence variation of the locus (other trans factor may bind here) but not only because of BZR1 binding to the locus.

4.As we all know that binding of TF to its targets are spatiotemporal, it is a bit hasty to say that the binding of BZR1 to its allele specific targets in 26-day-old seedling (mainly meristem) would lead to trait diversity at other developmental stages.

Minor concerns:

1. What is the biological significance of the binding of BZR1 to enhancer regions? Would be nice if the author could discuss a bit on this observation. By the way, how many BZR1 binding sites are locating in open chromatin?

2. Line 174-176 the uniquely mapped reads were used to quantify the relative haplotype binding. However, the reads mapped to both of the genomes (B73 and Mo17) should be also considered as binding targets of BZR1.

3. Regarding the conclusion that binding of BZR1 to DNA is DNA methylation related, experimental evidence is needed.

**Authors Response**

**Point-by-point responses to the reviewers’ comments:**

Reviewer #2: In this revised manuscript, the authors have conducted additional experiments and tried their best to fully address the reviewers' constructive comments, and as a result, the quality is substantially improved and the conclusions are much more solidified. This reviewer appreciates the authors' substantial efforts made to this work. Although some potential pitfalls are still hard to overcome at the current stage, I am satisfied with this revision and it does provide new insights into BR biology in maize.

A few minor suggestions:

1. The sentence in Abstract (Background) "Genetic variation in the BR-responsive cis-element is likely causal to trait variation" could be changed to "Genetic variation in the BR-responsive cis-element likely contributes to trait variation";

*Response: We agree and have changed the sentence accordingly.*

2. For RNA-seq, why shoot tissues were used? Explain

*Response: We analyzed BZR1-GFP activity and found that young shoot tissue showed high activity for BZR1, consistent with previous reports (Supplemental Fig 1). Hence, ChIP, RNA-seq, and HASCh-seq were performed in similar shoot tissue. The exception was previously published B73 & Mo17 inbred RNA-seq data which came from root tissue, yet it had the highest depth and quality so we thought it to be representative. Our own RNA-seq data in the F1 hybrid used more similar shoot/leave tissue.*

3. The statement of B73 has the best genome annotation might not be true anymore with the release of a number dozens of new maize genomes recently (Like Hufford et al., Science, 2021). Please double check.

*Response: For the best utility of our data we actually use the newest B73 version provided by Hufford et al., Science, 2021. We agree that a better statement is: “with the B73 inbred line, which is among the most well-studied and annotated maize lines (citing “Hufford et al., Science, 2021).”*

4. In a number of places, "F1" should be corrected to "F1 (lower case)".

*Response: We replaced F1 with F1.*

7. Do B73 and Mo17 show trait variation to NLB/SLB resistance/response? Or maybe they can also look at other founder lines of the NAM population to link ASBs to gene expression and trait variation?

*Response: Yes, multiple studies found a significant difference in NLB and SLB resistance between B73, Mo17, and NAM lines, too. We added “Considering the differences observed between B73, Mo17, and other NAM founders in their resistance to NLB and SLB [36,38,39], these ASBs linked to GWAS hits provide strong candidates for future functional studies.*

Do any of the ASBs co-localize or close to any QTLs for NLB/SLB resistance?

*Response: Yes. Quote from the manuscript: “we identified 11 ASBs which co-localized (within 2 kbp) with NLB/SLB GWAS hits [2]. We found that these ASBs, and their nearby genes, also co-localized with joint-linkage mapping QTLs for NLB and SLB in the NAM population (Fig. 5a).”*

Reviewer #3:

This study reported the allele specific binding profile of ZmBZR1 in a hybrid background. BZR1 was supposed to be key player in maize BR pathway. The author found that the binding affinity of BZR1 is DNA-methylation related and quite some binding sites locate in enhancer regions. More interestingly, they found a significant overlap between allele-specific binding of ZmBZR1 and trait related SNP in the GWAS analysis, indicating the importance of BZR1 binding preference in controlling trait performance. The work is novel in plants and brings the community a nice example to link TF binding and trait variation. However, there are some concerns need to be addressed.

Major concern:

1. The author documented quite some evidence to show that ZmBZR1 is a key regulator to control maize development, but they are indirect evidence. A knock-out mutant will be very important to enhance the biologically meaning of this work. It makes sense to say that the traits are affected by allele specific binding of BZR1 only when you see the trait is altered in the mutant. And, would be very nice to see that the mutant could be rescued by BZR1-GFP. Meanwhile, if a mutant is available, differentially expressed genes between wild type and the mutant would be more relevant to the binding of BZR1 then the BR induced genes.

*Response: As we previously pointed out, there is, unfortunately, no bzr1 mutant in maize. In Arabidopsis, the single bzr1 loss-of-function mutant shows no phenotype. Knocking out five BZR1 homologs resulted in dwarf and sterile phenotypes. In the absence of a quintuple mutant in maize, previous studies have provided many lines of evidence supporting the conserved function of ZmBZR1, the activity of its YFP fusion, and ZmBZR1-YFP’s expression in BR-responsive cells, as we discussed in the last paragraph of our introduction. Furthermore, we showed that BZR1-GFP’s nuclear accumulation is dependent on BR. We added in the introduction citations of more publications on rice BZR1: “Suppressing OsBZR family by RNAi leads to BR-insensitive phenotypes in rice, and expression of dominant OsBZR1S156G-GFP rescued Arabidopsis bri1 mutant, demonstrating the conserved function in monocots (Bai et al., 2007).” On the other hand, our study focuses on the genetic variation of cis-elements and epigenetic marks. The associations of these variations with traits are evidence for their function.*

2. Since the author performed ChIP in F1 background, in which two endogenous BZR1 copies are binding to their targets, better to verify that the binding affinity of BZR1-GFP is comparable to the endogenous ones (their genotype should be investigated). In this regard, complementation of bzt1 mutation by BZR1-GFP would help.

*Response: The presence of endogenous TF is not a problem for ChIP-seq experiments. In the literature, ChIP-seq experiments are mostly done in transgenic plants that express a tagged TF in the wild-type background where the endogenous TF competes for binding. The competition is expected to reduce the signal-noise ratio, but otherwise not to affect the binding sites detected by ChIP-seq.*

*In principle, there is a possibility that GFP affects the DNA binding affinity. However, our high-quality data prove that the BZR1-GFP fusion binds to its target cis-elements, the same as reported in Arabidopsis. This is consistent with the large body of literature that studied BZR1-GFP in Arabidopsis, rice, and maize, including ChIP-chip and ChIP-seq experiments. Therefore, all the evidence available so far in many studies in Arabidopsis, rice, and maize indicates that BZR1-GFP has the same function and DNA binding specificity as the endogenous BZR1. We found no amino acid difference in the BZR1 DNA binding domain between B73 and Mo17. We added: “The DNA-binding domain of BZR1 in B73 and Mo17 has an identical sequence (Supplementary Fig. S2), and therefore the variation of the BR response gene network should be due to variation of target DNA.” Our current study shows that variation in cis-elements contributes to phenotype variation.*

*The complementation of a mutant is a common test for the functionality of TF-GFP fusion. However, there is no maize bzr1 mutant nor Arabidopsis bzr1 mutant with a phenotype to complement. On the other hand, the functions of BZR1-GFP have been established in Arabidopsis and rice. We do not think that all the Arabidopsis and rice experiments should be repeated in maize, instead, we build on the knowledge gained in Arabidopsis and rice, and our results are consistent with findings in those species (i.e. enrichment of the BRRE element).*

*After all, the goal of our study is to identify variations in cis-element that affect ZmBZR1 binding. Even if BZR1-GFP has different DNA binding affinity from the endogenous BZR1, the allele specificity of binding should not be affected, and therefore, the ASBs should be relevant to the endogenous BZR1.*

3. line 243-250, the author showed that a certain proportion of genes with ASB showed allele-specific gene expression both between B73 and Mo17 and in B73xMo17 hybrids. However, this should be more due to the sequence variation of the locus (other trans factor may bind here) but not only because of BZR1 binding to the locus.

*Response: Yes, the allele-specific gene expressions were not all due to ASB, as 44.4% of the genes without ASBs also showed allele-specific expression. However, genes with ASBs are more likely to show allele-specific expression (55.2% vs 44.4% probability, p=0.0009). We carefully state: “Taken together these results suggest that variation of BZR1 binding contributes to variation of gene expression.” and do not overstate that BZR1 is acting alone.*

4.As we all know that binding of TF to its targets are spatiotemporal, it is a bit hasty to say that the binding of BZR1 to its allele specific targets in 26-day-old seedling (mainly meristem) would lead to trait diversity at other developmental stages.

*Response: We agree that TF binding to targets can vary spatiotemporally, e.g. through the abundance of the TF or changes in the chromatin. However, when mutations or epigenetic variations in stable marks such as DNA methylation turn a target into a non-target, developmental stages will not reverse such variations. Thus, it is to be expected that affinity differences observed between the two alleles affect the target genes whenever their regulation via BZR1 is relevant. The statistical correlations between ASBs and traits at other developmental stages are consistent with such an expectation.*

Minor concerns:

1. What is the biological significance of the binding of BZR1 to enhancer regions? Would be nice if the author could discuss a bit on this observation.

*Response: Indeed, our finding of BZR1 binding to enhancer regions is interesting as this was not known before. We added in the discussion: “While promoters are defined by their proximity to genes, distant enhancer regions, although important for gene regulation, are more difficult to pinpoint, in particular in plants, as they often lack the specific marks often associated with enhancers in animals [citations]. Oka et al. recently identified over a thousand such putative enhancers in B73 using various histone marks, chromatin accessibility, and DNA methylation. The finding of ASB enriched in those enhancer regions provides evidence for BZR1 functions through enhancers, which is not known previously.”*

By the way, how many BZR1 binding sites are locating in open chromatin?

*Response: We added: “and about 65% (11232) overlapped with previously identified open chromatin regions [citation]”*

2. Line 174-176 the uniquely mapped reads were used to quantify the relative haplotype binding. However, the reads mapped to both of the genomes (B73 and Mo17) should be also considered as binding targets of BZR1.

*Response: We removed the shared reads based on the following consideration. First, the goal of Hasch-Seq is to identify allele-specific variation in BZR1 binding, and thus the shared reads should not be included in the quantitation. Second, the maize genome contains a high density of SNPs (average 1 SNP per 100 bp), and our fragment size averages 500 bp, and thus the shared reads are a small portion of the dataset. Third, the negative controls (without BZR1-GFP) were analyzed in the same way, which ensures the validity of the ASB peaks. We will provide peak data which includes shared reads in the supplement and for our planned upload to the MaizeGDB genome browser.*

3. Regarding the conclusion that binding of BZR1 to DNA is DNA methylation related, experimental evidence is needed.

*Response: Our discussion includes: “which in vitro has been shown to reduce the interaction of most TFs, including Arabidopsis BES1/BZR2, with their target sequence [49].” Our results strongly support these in vitro observations now in vivo. We previously stated that multiple attempts of in vitro DAP-seq assays (with and without native DNA methylation) for ZmBZR1 were undertaken but the results were inconclusive, which is consistent with previous results showing only a few binding peaks for AtBZR1 DAPseq (ReMAP2022 database https://remap2022.univ-amu.fr/).*

*As far as we know, our study is the first demonstration of the effects of DNA methylation on allele-specific TF binding in vivo in plants.*

**Third round of review**

**Reviewer 3**

The author nicely addressed most of my concerns but I still believe that precise estimation of the genetic effect of ZmBZR1 by mutant is important for this study. When correlating the binding with phenotypic variation, we have to consider how much phenotypic variation could be caused by the change of binding motif of a single gene, even it is a transcription factor. Nevertheless, this work offers the first example to link ASB with genetic variation, which is a new addition to the field. I would be happy to recommend it for publication.
